# Intimate Interaction Between Nucleic Acid and Conjugated Polymers in Organic Electrochemical Transistors Enables Ultrasensitive Biomarker Detection

**DOI:** 10.1002/adma.73802

**Published:** 2026-06-25

**Authors:** Hong Liu, Naixiang Wang, Anneng Yang, Jiajun Song, Li Li, Iain McCulloch, Helen Ka‐wai Law, Feng Yan

**Affiliations:** ^1^ Department of Applied Physics The Hong Kong Polytechnic University Kowloon Hong Kong P. R. China; ^2^ Institute of Integrative Systems and Design The Hong Kong University of Science and Technology Kowloon Hong Kong P. R. China; ^3^ Department of Chemistry Chemistry Research Laboratory University of Oxford Oxford UK; ^4^ Department of Electrical and Computer Engineering Andlinger Center for Energy and the Environment Princeton University Princeton NJ USA; ^5^ Department of Health Technology and Informatics The Hong Kong Polytechnic University Kowloon Hong Kong P. R. China; ^6^ Research Institute of Intelligent Wearable Systems The Hong Kong Polytechnic University Kowloon Hong Kong P. R. China

**Keywords:** biosensors, organic electrochemical transistors, RNA detection, volumetric capacitance

## Abstract

Ultrasensitive biosensors are crucial for pandemic prevention and healthcare monitoring. The interactions between biomolecules and functional materials, such as metals and semiconductors, have led to the development of highly sensitive biosensors. Organic semiconductors, known for their unique mixed ion and electronic conduction properties in aqueous solutions, show promise in these applications. However, the interactions between organic semiconductors and biomolecules remain poorly understood. In this study, we investigate the interaction between nucleic acid and a conjugate polymer by analyzing the performance of organic electrochemical transistors (OECTs). For the first time, we demonstrate that negatively charged ribonucleic acid (RNA) can interact with the conjugate polymer, leading to a decrease in the volumetric capacitance of the polymer channel in aqueous solutions. Based on this effect, OECTs are employed to detect various RNA biomarkers with high sensitivity, achieving multiplexed and selective detections of multiple RNA biomarkers at a low detection limit down to 10^−17^ M. This portable biosensing platform can facilitate rapid, low‐cost and point‐of‐care diagnostics for various diseases.

## Graphical abstract

The intimate interaction between negatively charged RNA molecules and conjugated polymers in organic electrochemical transistors (OECTs) in aqueous electrolytes is investigated. It demonstrates that RNA binding reduces the volumetric capacitance of the polymer channel, enabling the development of an ultrasensitive biosensing platform capable of multiplexed RNA biomarker detection down to 10^−^^1^ ^7^ M for point‐of‐care diagnostics.

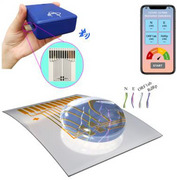

## 1 Introduction

Organic bioelectronics is a highly interdisciplinary field and has attracted much research interests because it can bridge the communication between electrical and biological signals via both electrons and ions [1, 2, 3]. Since the first demonstration of conducting polymer by Shirakawa et al. in 1970s [4], organic semiconductors have been functionalized and utilized in various bioelectronic devices with the advantages of low cost, solution processability, good mechanically flexibility, tunable properties via molecular design and biocompatibility [5, 6, 7]. It is convenient to integrate organic electronic devices into biological systems and obtain bio‐signals based on the interactions between biomolecules and the organic devices [8, 9, 10, 11]. This type of interactions could be ion exchange, electrochemical reaction or surface potential modification on the device surfaces in aqueous solutions [12, 13, 14, 15]. Because of the complex and dynamic morphology of organic semiconductors and biomolecules in aqueous solutions, directly characterizing their interactions using conventional techniques poses significant challenges.

Organic electrochemical transistors (OECTs) based on specific conjugated polymers, such as poly(2‐(3,3’‐bis(2‐(2‐(2‐methoxyethoxy)ethoxy)ethoxy)‐[2,2’‐bithiophen]‐5‐yl)thieno[3,2‐b]thiophene) (p(g2T‐TT)), poly(3,4‐ethylenedioxythiophene)‐poly(styrenesulfonate) (PEDOT:PSS) and poly([N,N’‐bis(2‐octyldodecyl)‐1,4,5,8‐naphthalene dicarboximide‐2,6‐diyl]‐*alt*‐5,5’‐(2,2’‐bithiophene)) p(NDIOD‐T2) [16, 17, 18], show excellent transistor performance, especially high transconductance, and have been successfully employed for numerous highly sensitive biosensors [19, 20, 21, 22]. Due to their inherent amplification function, OECTs display channel currents sensitive to biomolecules immobilized on their surfaces [23, 24, 25], while the interaction between biomolecules and organic semiconductors remains underexplored. Here, we investigate the interaction between ribonucleic acid (RNA), a negatively charge biomolecule, and an organic semiconductor p(g2T‐TT) in aqueous solutions by characterizing the performance of OECTs [26]. When the p(g2T‐TT) channel of an OECT is modified with RNA molecules, we find that the RNA molecules impede the bulk doping of p(g2T‐TT) in aqueous electrolytes, resulting in an obvious decrease in the volumetric capacitance of the OECT channel.

Considering biosensors leveraging interactions between target molecules and semiconductors in thin‐film transistors usually exhibit high detection sensitivity [27, 28, 29, 30], we then developed label‐free RNA sensors based on the interactions between RNA biomolecules and organic semiconductors in OECTs. RNA holds promise as a valuable biomarker for the diagnosis and treatment of various diseases, including cancers and virus infections [31]. Conventional RNA sensing techniques are mainly based on quantitative polymerase chain reaction, northern blotting and complex optical techniques, which highly rely on expensive large equipment or time‐consuming functionalization processes with fluorescence labels [32, 33]. Several label‐free and portable techniques have been developed for RNA analysis, such as microcantilevers [34], surface plasmon resonance [35], and electrochemical detections [36]. However, they are normally not sensitive enough for practical applications. In this work, OECTs are successfully used to detect different RNA biomarkers at low concentrations with high selectivity. Furthermore, a portable sensing system based on an OECT array is developed, and several RNA sequences from severe acute respiratory syndrome coronavirus 2 (SARS‐CoV‐2) are selectively detected, demonstrating the capability of the sensing system for multiple RNA sequence screening, which can improve diagnosis speed and accurary of diseases.

## 2 Results and Discussion

### 2.1 Flexible OECTs for RNA Sensing

**FIGURE 1:**
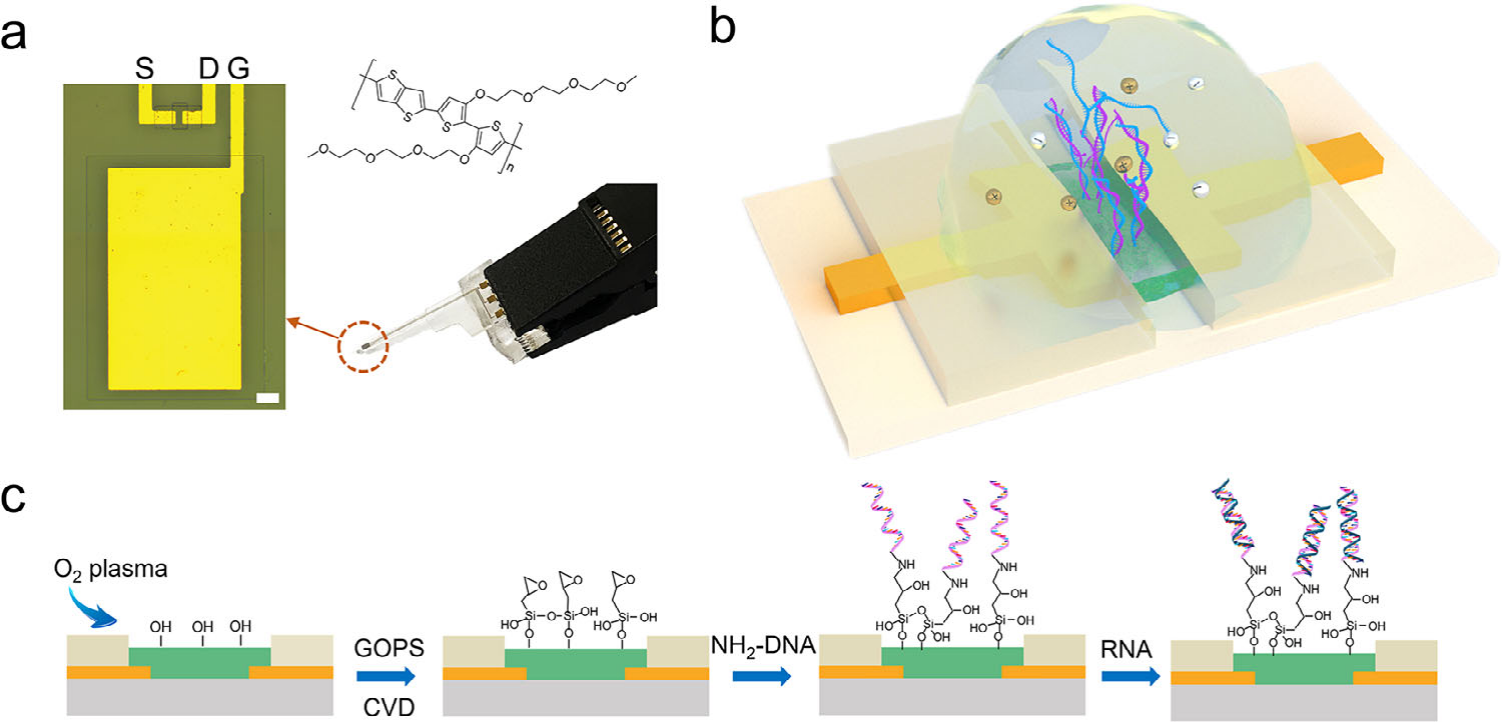
Schematic diagram of the p(g2T‐TT)‐based OECT and functionalization procedures for RNA detection. (a) Optical images of an OECT with the p(g2T‐TT) active layer; Scale bar in the image is 100 µm. (b) Schematic of the OECT biosensor for RNA sensing; (c) Functionalization procedures of the OECT for capturing RNA molecules.

As illustrated in Figure 1a, flexible OECTs with Au electrodes and p(g2T‐TT) active layers were prepared on thin polyethylene terephthalate (PET) substrates (thickness: 200 µm) by a photolithography microfabrication process [37, 38]. The active layer thickness of the devices was initially regulated to approximately 100 nm. The source and drain electrodes were well protected from the electrolyte by the coverage of SU‐8 photoresist insulation layer, leaving the channel area exposed to the electrolyte. The devices can be easily bent to various statuses, as shown in Figure S1a. The transfer characteristic (I_D_ vs. V_G_) of a device was measured in 0.1X phosphate buffered saline (PBS) solution at the drain voltage V_DS_ = −0.5 V. The device is flexible and can show stable performance at different bending status. As shown in Figure S1b, the OECT can operate in the p‐channel accumulation mode with stable performance at different bending radii (r = 0.5, 1, 2 cm and ∞).

Figure 1b shows the schematic diagram of an OECT for immobilizing RNA on the organic channel. The miRNA‐21 with the sequence of 5’‐U AGC UUA UCA GAC UGA UGU UGA‐3’ was first chosen as the detection target in the experiment. Notably, miRNA‐21 has been widely implicated in cancer pathogenesis, with dysregulation observed in over ten cancer types, including breast, colorectal, lymphoma, and ovarian tumors [39]. To facilitate an interaction between target RNA and the organic channel, probe deoxyribonucleic acid (DNA) was immobilized on the channel surface to capture the target RNA. The functionalization procedure is illustrated in Figure 1c. First, the p(g2T‐TT) film was treated with oxygen plasma for 2 min to generate surface hydroxyl groups. Subsequently, a (3‐glycidyloxypropyl)trimethoxysilane (GOPS) monolayer was deposited via chemical vapor deposition (CVD) as previously reported [40]. After that, amino‐modified single‐strand DNA probes are immobilized on the channel surface through chemical bonding to the epoxide group on GOPS, enabling the capture of complementary target RNA molecules in solution. The probe sequence (5’‐NH2‐C6‐TCA ACA TCA GTC TGA TAA GCT A‐3’) is complementary to miRNA‐21, where G, C, A, and T denote guanine, cytosine, adenine, and thymine, respectively. Successful functionalization was verified by fluorescence microscopy using fluorescein isothiocyanate‐labeled polyethylene glycol amine (FITC‐PEG‐NH2) (Figure S2).

**FIGURE 2:**
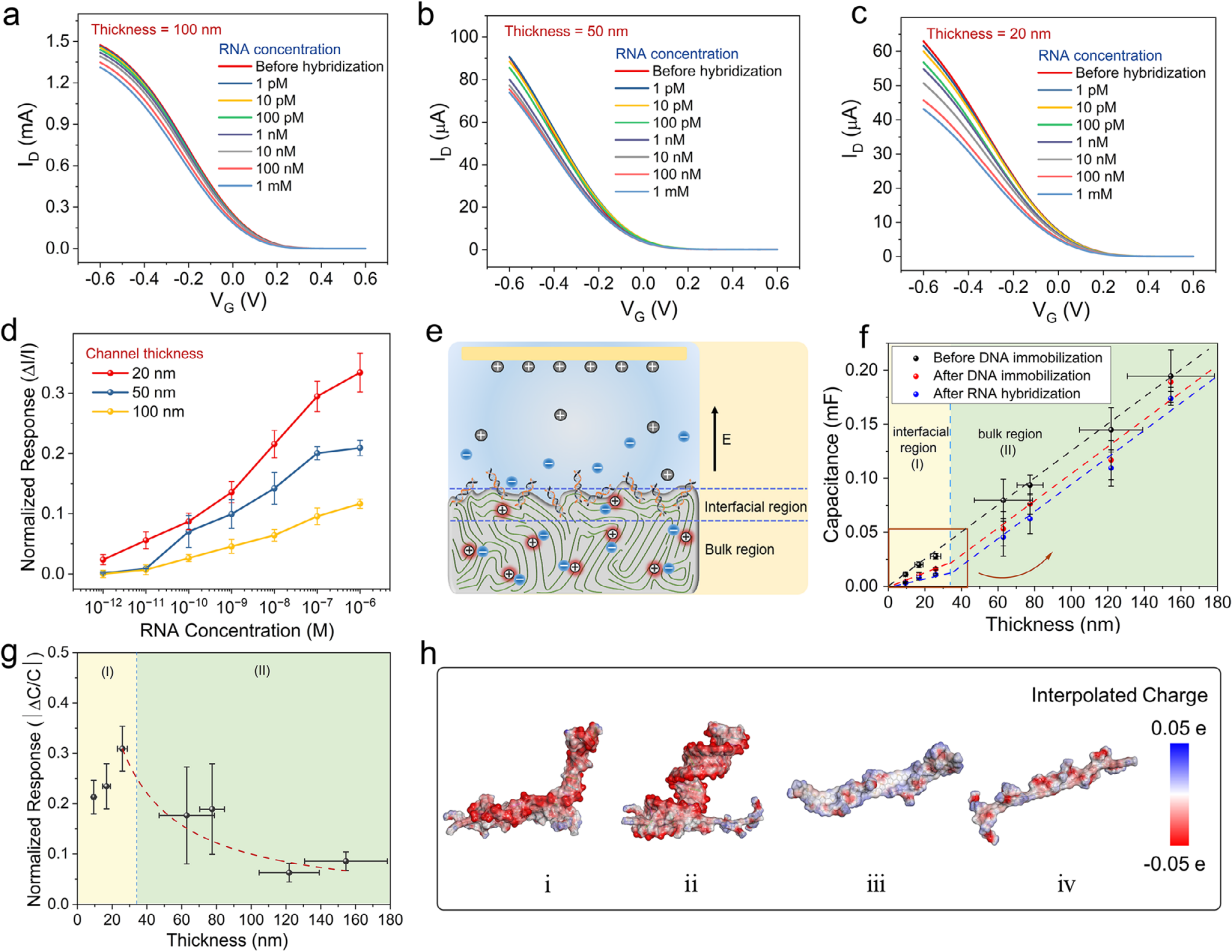
The performance of OECT‐based RNA sensors with different channel thicknesses. The transfer curve variations of the OECT‐based RNA sensor with p(g2T‐TT) films of different thicknesses: (a) 100 nm, (b) 50 nm, and (c) 20 nm, upon exposure to increasing concentrations of complementary miRNA molecules. (d) Normalized current decreases of OECTs with different channel thicknesses (100, 50, and 20 nm) as functions of RNA concentration. V_DS_ = −0.5 V. Error bar was calculated based on the responses of at least 3 identical devices. (e) Schematic of the sensing mechanism illustrating the interaction between RNA and polymer in the channel area of an OECT; (f) Effective capacitance before DNA immobilization (black), after DNA immobilization (red) and after RNA hybridization (blue) for p(g2T‐TT) films coated on ITO substrates with varying film thickness; (g) Normalized capacitance response vs. p(g2T‐TT) film thickness. Red dash line indicates the fitting of the average relative capacitance change reversely proportional to the film thickness. The average value and error bar were calculated based on the responses of at least 10 devices prepared at the same conditions. (h) Interpolated charge of (i) polymer‐DNA, (ii) polymer‐DNA‐RNA complex and polymer (iii) before and (iv) after the hybridization of RNA extracted from molecular dynamics simulations.​.

Following DNA probe functionalization, device performance was characterized in PBS solution before and after a 1‐h RNA hybridization incubation to assess the impact of RNA capture. As shown in Figure 2a, channel currents exhibited a concentration‐dependent decrease across RNA concentrations ranging from 1 pM to 1 µM. Notably, the maximum current at V_G_ = −0.6 V was decreased for up to 10% while the threshold voltage (Figure S3) has no change. The unchanged threshold voltage indicates that the doping level of p(g2T‐TT) is not influenced by the negatively charged RNA molecules in PBS solutions. Therefore, the observed current reduction upon RNA exposure can be primarily attributed to the decrease in the OECT transconductance.

Considering that the chemical modification should occur on the channel surface, RNA was supposed to be captured primarily on the p(g2T‐TT) channel surface, affecting only near‐surface channel current. To amplify the RNA sensing effect, we systematically reduced the channel thickness to 50 and 20 nm (Figure 2b,c). The 20 nm‐thick channel exhibited the most pronounced response, with channel currents decreasing by ∼31% after 1 µM RNA capture (Figure 2c). These results demonstrate that surface‐bound RNA molecules exert a stronger influence on channel current in thinner semiconductor layers.

Figure 2d summarizes the statistically normalized responses of devices to RNA concentrations ranging from 1 pM to 1 µM. The effect of channel thickness on the relative change of current response can be clearly observed. The device with a channel thickness of 20 nm exhibits a clear channel current change when the RNA concentration is down to 1 pM while the devices with thicker channels (50 and 100 nm) can only have responses to RNA at 100 pM. These results confirm that reduced channel thickness amplifies the effect of RNA molecules on channel properties, thereby enhancing detection sensitivity. Based on these findings, all subsequent experiments employed 20 nm‐thick channels unless otherwise specified.

The modification of RNA molecules on a p(g2T‐TT) channel of an OECT is illustrated in Figure 2e. When a negative V_G_ is applied on the gate, anions (mostly chloridion) are driven to penetrate into the p(g2T‐TT) film and corresponding holes on the conjugated backbone of the polymer chains are generated through the bulk doping process [41]. Therefore, the channel current in the saturation regime is given by [42]:

(1) $$I_{D}=\frac{W}{2L}\;\cdot \mu \cdot C_{i}\cdot \;{\left(V_{th}-V_{G}\right)}^{2}=\frac{W}{2L}\;\cdot \mu \cdot d\cdot C^{*}\cdot {\left(V_{th}-V_{G}\right)}^{2}$$

where *W* and *L* are the width and length of channel, *µ* is the hole mobility, C^*^ is the volumetric capacitance, *V*_th_ is the threshold voltage, *d* is the thickness of channel layer, and *C*_i_ is the capacitance per unit area.

According to Equation (1), the decrease in drain current induced by RNA molecules might be related to the variation of the mobility *µ* or the capacitance C_i_ of the device because V_th_ is not influenced by RNA binding. Therefore, electrical impedance spectroscopy (EIS) characterization was carried out to investigate the effect of RNA hybridization on the surface of p(g2T‐TT) films. Figure 2f,g show the effective capacitance changes of p(g2T‐TT) films with various thicknesses (from 10 to 154 nm) before and after the immobilization of DNA probes and the hybridization of RNA. The concentration of RNA for hybridization is 1 µM for all samples. The EIS was measured under an effective constant voltage bias of −0.6 V, which was equal to the maximum gate voltage in OECTs. Figure 2f reveals distinct capacitive behaviors between bulk (>35 nm) and interfacial (≤35 nm) regions. For bulk films, capacitance shows a parallel decrease following DNA immobilization, with further reduction after RNA hybridization. This contrasts sharply with interfacial films, producing a characteristic kink at the 35 nm transition boundary. Notably, RNA hybridization induces a maximal 31% normalized capacitance decrease in ∼20 nm films (Figure 2g), with the effect diminishing to <10% for thicknesses exceeding 100 nm. These results demonstrate that DNA immobilization and RNA hybridization modulate the volumetric capacitance of p(g2T‐TT) films exclusively within the interfacial region. When the film thickness is less than 35 nm, DNA and RNA on the surface can influence the whole volumetric capacitance of the films presumably due to the porous structure of the films. Therefore, the change of capacitance (*ΔC*) is proportional to the film thickness *t* (*ΔC* ∝ *t*) and the relative change of the capacitance (*ΔC/C*) is almost constant. When the thickness of the p(g2T‐TT) film is larger than 35 nm, the DNA and RNA can only influence the volumetric capacitance of the film in the fixed interfacial region and thus the change of capacitance is almost constant for different p(g2T‐TT) film thickness. In this case, the relative change of capacitance is reversely proportional to the film thickness (*ΔC/C* ∝ *1/t*, the dash line in region (II) in Figure 2g). Therefore, the RNA molecules hybridized at the surface of a p(g2T‐TT) film could only affect the interfacial region of the film. This result is consistent with the sensor response of OECTs with different channel film thicknesses as previously discussed. In the OECT with a 20‐nm thick channel, the whole p(g2T‐TT) film is in the interfacial region, hence RNA molecules have the largest influence on the device performance and the device exhibits the highest sensitivity to RNA.

The normalized capacitance changes of a 20 nm‐thick p(g2T‐TT) film as a function of RNA concentration during modification is calculated (Figures S4 and S5). The capacitance decreased by up to 30% at 1 µM RNA concentration, which is consistent with the OECT response to RNA. A control experiment was carried out to test non‐complementary RNA solution, showing a less than 4% capacitance decrease (Figure S5). Furthermore, the carrier mobilities of the OECTs under different RNA concentrations can be calculated from the saturation regime of the transfer curves according to Equation (1). We can find that the carrier mobility of p(g2T‐TT) is hardly influenced by RNA on the surface (Figure S6). The results indicate that the capacitance change is the major reason for the decrease of the channel current during the RNA sensing process.

The detailed bulk doping process in p(g2T‐TT) chains is illustrated in Figure S7a. Under a negative V_G_, anions (i.e. Cl^−^) in the electrolyte are driven by the applied electric field to migrate toward the p(g2T‐TT) chains and generate considerable amounts of holes in the conjugated backbone of the polymer, leading to a high volumetric capacitance in the p(g2T‐TT) film [16]. When target RNA molecules are captured by the complementary DNA probes, they can move for a certain distance limited by the length of grafted GOPS and the length of RNA molecules (7∼ 10 nm) [43]. As reported recently, ions can diffuse into not only the bulk of a p(g2T‐TT) polymer film but also polymer crystallites [41]. However, the RNA molecules cannot diffuse into the polymer crystallites because of their large molecular size [44]. Considering the fact that RNA molecules possess large amounts of phosphate groups and a relatively low isoelectric point (∼2) [45], the captured RNA molecules have high density of negative charges in the PBS solution, which can prohibit the diffusion of other small anions from the aqueous electrolyte to p(g2T‐TT) chains. For probe DNA molecules, the isoelectric point is near 5 [46], so the effect should be weaker. As illustrated in Figure S7a (right figure), within the electrostatic screening length in PBS solution, some chloride anions are electrostatically repulsed by negatively charged RNA strands attached on p(g2T‐TT) chains, which is similar to the effect of a polymer/RNA complex system reported before [47]. As a result, the RNA molecules immobilized on the p(g2T‐TT) chains can substantially reduce the effective surface area of p(g2T‐TT) chains for bulk doping and decrease the volumetric capacitance C^*^ of the film.

The interaction between captured RNA molecules and a p(g2T‐TT) film was further investigated by using Fourier transform infrared spectroscopy (FTIR) spectroscopy with attenuated total reflectance (ATR) module. The FTIR spectra of the pristine p(g2T‐TT) film and the films with the modification of GOPS, single‐strand DNA, and hybridization of RNA molecules are presented in Figure S7b. The dominant spectral band centered between 1070 and 1101 cm^−1^ was assigned to the C─O─C stretching vibration from the glycolated side chain of p(g2T‐TT) [48]. Notably, the absorption peak was not influenced by the modification of GOPS. However, a shift from 1070 to 1043 cm^−1^ and broaden of the absorption band was observed after the immobilization of DNA on the surface. A further shift to 1029 cm^−1^ was observed after the hybridization of RNA. This effect could be ascribed to the formation of hydrogen bonds between the glycolated side chain of p(g2T‐TT) and DNA/RNA molecules. As illustrated in Figure S7a, the oxygen atoms from glycolated side chains form N─H─O hydrogen bonds with the base groups from the nucleotide structure. The stretching force constant for C─O─C could be changed after the formation of hydrogen bonds, resulting in a shift of the absorption peak to lower frequencies and a broader band, further reflecting the influence of nucleic acid functional groups on the polymer [49]. This result indicates that intimate interactions between RNA and p(g2T‐TT) chains exist in RNA detections. So polyanionic RNA molecules covered on the p(g2T‐TT) chains closely can reduce the effective surface area for bulk doping.

To further confirm and visualize the effect of captured RNA helix on p(g2T‐TT) chains, a cylinder model is presented to simulate the capacitance changes of p(g2T‐TT) (Figure S8, Note S1). When the distance of p(g2T‐TT) and RNA helix is reduced to 4.1 nm, a 30% capacitance decrease is calculated for p(g2T‐TT) film, which is consistent with the experimental results from OECT biosensors and p(g2T‐TT) films. Besides, AC transconductance (g_m_ = ΔI_D_/ΔV_G_) was characterized on the OECTs to investigate whether the RNA molecules have an impact on anion penetration speed from the PBS solution to the polymer film [37]. The phase shift angle of the transconductance as a function of frequency was recorded to present the dynamic response of an OECT before and after the capture of RNA molecules. As shown in Figure S9, with the increase of RNA concentrations, the phase angles of transconductance curves almost overlap, implying that transient characteristics are hardly changed. This result indicates that the existence of RNA molecules on the surface of p(g2T‐TT) film do not influence the diffusion speed of anions into the bulk of the film.

To mimic the effect of RNA molecules on the capacitance of p(g2T‐TT), we mixed negatively charged polystyrene sulfonate (PSS–) or neutral poly(vinyl alcohol) (PVA) macromolecules into PBS solutions to characterize the volumetric capacitance of a p(g2T‐TT) film. The PSS^–^ and PVA have similar average molecular weight of around 100 kDa and sizes comparable to that of the RNA molecules. The macromolecules therefore cannot diffuse into the p(g2T‐TT) crystallites but can cover the surface of the polymer chains. The concentrations of PSS^–^ and PVA were increased from 0.005 to 0.5 M (calculated based on repeat units) while the concentration of other ions in the PBS solution were fixed. The EIS of the p(g2T‐TT) film was characterized in the mixture solutions under a bias voltage of −0.6 V. As shown in Figure S10, the capacitance of p(g2T‐TT) film decreases for about 30% when the concentration of PSS is 0.5 M in PBS solution, while for the same concentration of PVA added in PBS solution, only a less than 10% decrease can be observed. This effect could be attributed to the negative change of PSS^–^ in the aqueous solution. Under the negative bias voltage applied between the counter electrode and the p(g2T‐TT) film (−0.6 V), negatively charged PSS^–^ in the PBS solution can move toward the p(g2T‐TT) polymer chains and again prohibit other small anions to dope p(g2T‐TT) due to the electrostatic force, which is similar to the role of RNA on the surface of p(g2T‐TT) film discussed above. However, when PVA is mixed in the PBS solution, although PVA has the comparable molecular weight of PSS, much smaller capacitance change is observed because the neutral PVA chains could not effectively prohibit the diffusion of anions to the p(g2T‐TT) chains. This result provides compelling evidence that negatively charged macromolecules including RNA can decrease the volumetric capacitance of a conjugated polymer by repelling anions around the polymer chains via coulombic repulsion.

### 2.2 Molecular Dynamics Simulations of Polymer‐Nucleic Acid Interactions

Atomistic molecular dynamics simulations were performed to reveal the interactions between polymer and DNA/RNA. The hybridization of RNA with DNA probe promotes tighter association between polymer and nucleic acids. This leads to a more compact structure hinders the penetration of water molecules and ions into the polymer matrix, which in turn results in a reduction of the volumetric capacitance of the channel after the RNA hybridization (Notes S2 and S3, Figures S11 and S12, and Table S1).

Furthermore, to investigate the effect of RNA hybridization on the electrostatic properties of the polymer film, we performed molecular surface electrostatic potential analysis for both the polymer‐DNA and polymer‐DNA‐RNA complex. The Coulomb electrostatic potential on the surface of the polymer component was computed and visualized throughout the simulation trajectory. As shown in Figure 2h, significant differences in interpolated charge distribution were observed between the two complexes. After RNA hybridization, the spatial arrangement and intensity of positive/negative charges on the polymer surface were markedly reconfigured. Figure 2h(i,ii) display the overall interpolated charge distribution of the polymer‐DNA complex before and after RNA hybridization, respectively. The introduction of RNA notably increases the overall negative electrostatic potential of the polymer‐DNA‐RNA complex. Figure 2h(iii,iv) respectively show the interpolated charge distribution on the polymer surface before and after RNA hybridization, indicating that RNA incorporation enhances the area of negative potential on the polymer surface. The increased negative potential on the polymer surface can effectively suppress anion doping into the polymer film, thereby reducing the volumetric capacitance of the channel.

### 2.3 Performance of OECT‐Based RNA Sensors

Based on the intimate interaction between RNA and p(g2T‐TT) molecules, we then realize label‐free RNA sensors using OECTs. Notably, the selectivity or specificity of the sensor is crucial to its practical applications. To verify the sensing selectivity, we tested a single‐base mismatched RNA, a three‐base mismatched RNA, and a non‐complementary RNA with the OECT biosensors. The RNA molecules with different concentrations up to 1 µM were measured with the same procedure. As shown in Figure S13a, the maximum channel current of the RNA sensor was reduced by 11% when the device was exposed to the single‐base mismatched RNA, while the currents were hardly changed by three‐base mismatched and non‐complementary RNA, as illustrated in Figure S13b,c. The normalized current responses induced by these RNA molecules and the fully complementary RNA targets are summarized in Figure S13d, indicating excellent selectivity of the RNA sensor.

To further demonstrate the specificity of the RNA sensors, Hela cancer cell and adenocarcinomic human alveolar basal epithelial (A549) cell lines were cultivated and tested by using the devices (Figure S14a). A549 cell lines are normally used as models for studying lung cancer. As reported in the literature, the expression level of miRNA‐21 in A549 cells is much higher than that in Hela cancer cells [50, 51]. The two cancer cell lines were cultured with increasing cell numbers from 10^4^ to 10^6^. Then the samples for RNA detections were obtained with commercialized PureLink miRNA Isolation Kits. As shown in Figures S14b and S15, the sensor response suggests a significant overexpression of miRNA‐21 in A549 cells and the response increases with the increasing number of cells from 10^4^ to 10^6^ during cell culture. On the contrary, the blank sample and Hela cells exhibit no significant response compared to A549, indicating relatively lower expressions of miRNA‐21 in Hela cells, even at the condition of high cell numbers. The results demonstrate the great potential of using the label‐free OECT‐based sensing platform for in vitro biomedical applications.

**FIGURE 3:**
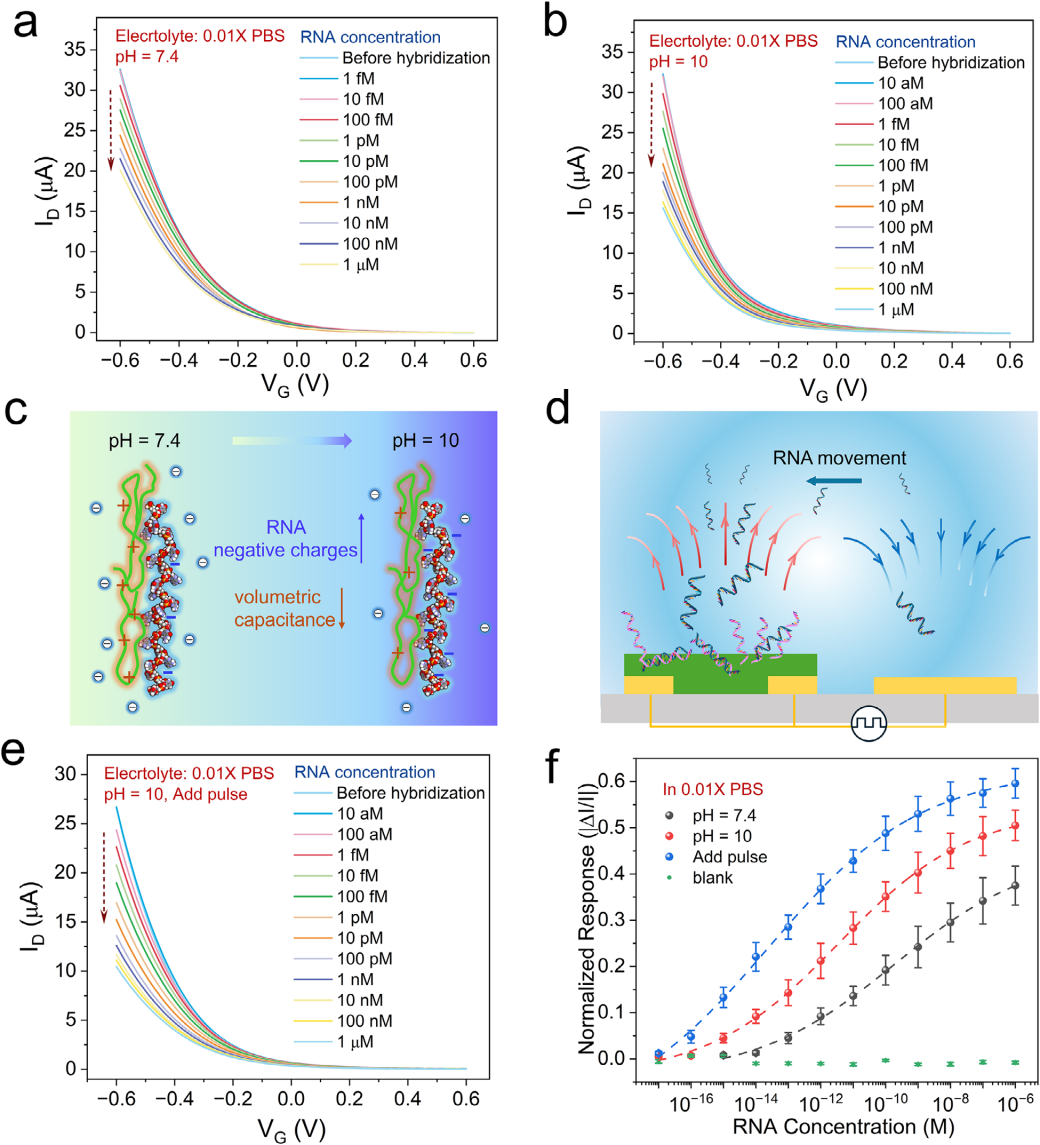
Responses of OECT‐based RNA sensors under different testing conditions. (a) The transfer curve variations of the RNA detection in diluted electrolyte (0.01X PBS, pH = 7.4). (b) The transfer curve variations of the RNA detection in alkaline electrolyte (0.01X PBS, pH = 10). (c) Schematic illustration of pH‐dependent charge concentration modulation in the channel (volumetric capacitance was decreased). (d) Schematic illustration of electric field‐enhanced RNA transport during pulse application. (e) The transfer curve variations of the RNA detection after applying voltage pulses during RNA hybridization. (f) Comparative analysis of normalized current response reduction across different optimization parameters.

To achieve a lower detection limit and higher sensitivity for RNA, the device response was amplified through (1) increasing the Debye length at the channel interface and (2) amplifying the negative charge density of RNA molecules during the test process. First, the electrolyte concentration was reduced to 0.01X PBS, extending the Debye length to ∼7 nm to fully cover single‐stranded RNA. As shown in Figure 3a, this improvement increased the current response amplitude, generating a 4.8% average current change at 100 fM RNA concentration with a background noise of 1.2% (signal‐to‐noise ratio > 3). Second, considering RNA's low isoelectric point (pI ∼2), we increased electrolyte pH to boost RNA charge density in the electrolyte during the test process. The stability of p(g2T‐TT) OECTs was initially assessed in electrolytes with pH values ranging from 9 to 12. Stable operation was consistently observed at pH ≤ 10 (Figure S16). Comparative testing at different pH levels confirmed that electrolyte with higher pH value enhances detection sensitivity (Figure S17). As demonstrated in Figure 3b, higher pH value of test electrolyte lowered the RNA detection limit to 1 fM, with a corresponding current decrease of around 4.5%. Also, the current response increased to 50.5% at 1 µM RNA concentration. Figure 3c schematically illustrates the mechanism by which an increase in the RNA surface negative charge leads to a reduction in the volumetric capacitance of the polymer.

To enhance detection sensitivity and accelerate DNA‐RNA hybridization, a voltage pulse (−0.5 V, 1 kHz) was applied during the hybridization process for 10 min, as illustrated in Figure 3d. The applied voltage pulse significantly improved hybridization efficiency and speed, leading to a more substantial current reduction and a lower detection limit (Figure 3e). Figure 3f presents the normalized current decreases of OECTs under different test conditions, including reduced electrolyte concentration, increased the pH value of electrolyte, and the application of a voltage pulse during hybridization. Through optimization of both testing condition and reaction parameters, the RNA detection limit was reduced by several orders of magnitude to aM level. When detecting RNA at varying concentrations, we used RNA‐free PBS solution as the control and performed measurements at 1‑h intervals. The results demonstrate good device stability, with signals consistently remaining within ±1.2% of the background level. The test results in Figure 3f were fitted with a dose‐response model, showing excellent fit (R^2^ > 99% for all curves). Based on the fitted curves, the limit of detection (LOD) for the miRNA was calculated to be 35 aM. Detailed fitting procedures are provided in the Supporting Information and Figure S18. Comparative studies using the gate electrode to capture RNA on the surface via hybridization under identical conditions (0.01X PBS, pH = 10) yielded a detection limit of 0.1 pM (Figure S19), demonstrating superior performance when employing the organic channel as the biorecognition site.

### 2.4 Portable and Multiplexing Detections of COVID‐19 RNA

A selected sequence of open reading frame 1ab (ORF1ab) gene, which has been confirmed to be a signature sequence presented in the COVID‐19 virus [52], was also detected using this OECT biosensor. The amino functional group‐modified complementary DNA probe with a sequence of 5’‐ NH_2_ – C6 – C CAT AAC CTT TCC ACA TAC CGC AGA CGG – 3’ was first immobilized on the channel area of an OECT sensor following the same procedure as previously described. Then the target ORF1ab RNA with a characteristic sequence of 5’—CCG UCU GCG GUA UGU GGA AAG GUU AUG G – 3’ was measured under the same condition. The transfer characteristics and the corresponding normalized responses to RNA concentrations are shown in Figure S20. Like our previous devices, the RNA sensors show a detection limit down to 76 aM, and the normalized current response is higher than 50% when the ORF1ab RNA concentration is 1 µM. Meanwhile, in the control experiments, the non‐complementary RNA molecules lead to a much lower device response, demonstrating the high selectivity of the label‐free RNA sensors.

**FIGURE 4:**
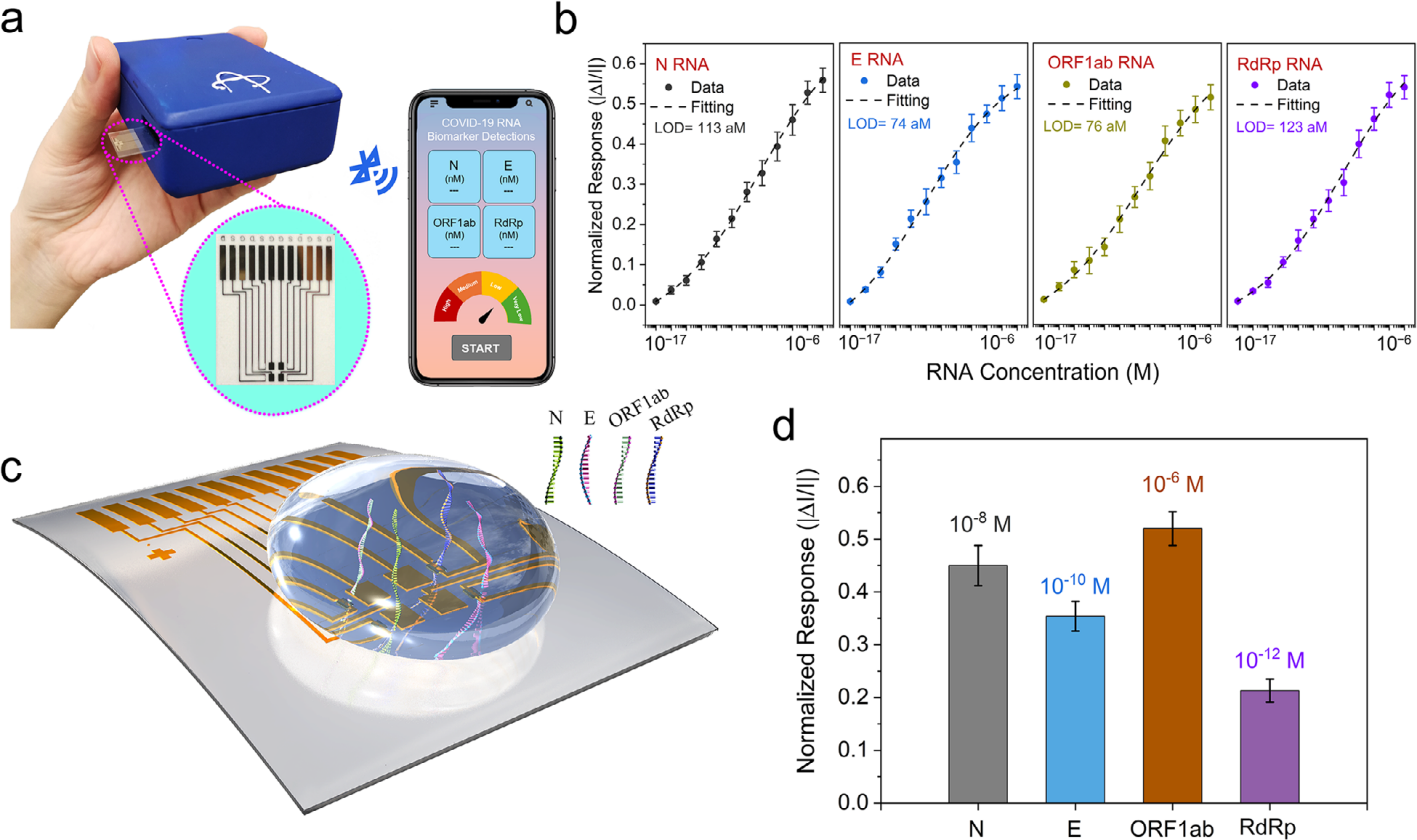
Multiplexed RNA detections using OECT‐array sensors. (a) Integrated detection system showing the flexible OECT array connected to a portable reader with Bluetooth‐enabled smartphone interface. (b) Normalized current response of OECTs as functions of the concentrations of selected COVID‐19 RNA sequences (N, E, ORF1ab and RdRp). V_DS_ = −0.5 V, the dashed line is the dose‐response fitting curve. (c) Schematic diagram of the OECT array for detection of multiple RNA sequences in single aqueous droplet. (d) Normalized current response of the OECT sensor array to different selected sequences with various concentrations. Error bars correspond to the responses measured from 3 different devices.

For multiplexed detection, a flexible OECT array was fabricated comprising four independent devices on a PET substrate. Each device can be functionalized with distinct DNA probes, enabling simultaneous detection of multiple RNA targets in a single sample. As illustrated in Figure 4a, the array responses are acquired by a portable meter and wirelessly transmitted to a mobile device via Bluetooth, demonstrating a complete point‐of‐care detection system. To enhance COVID‐19 diagnostic accuracy, we targeted four characteristic SARS‐CoV‐2 genomic sequences for multiplex detection: RdRp, ORF1ab, E, and N genes. This multi‐gene approach improves detection reliability by reducing false negatives from potential viral mutations [53]. The specific oligonucleotide probe sequences (Table S2) were designed against conserved regions of each target gene. As shown in Figure 4b and Figure S21, all of the RNA sequences lead to similar device response with the detection limit at the levels of tens of aM. Then a mixture solution of four types of RNA, containing N (10 nM), E (100 pM), ORF1ab (1 µM) and RdRp (1 pM), was prepared to simulate the actual sample analysis. As illustrated in Figure 4c, the sensor array was functionalized with four corresponding DNA probes on four independent channels in advance and then the mixture solution was dropped on the array channel for incubation for 10 min under voltage pulses. The device response is shown in Figure 4d, clearly distinguishing the concentration difference of the four sequences in one sample solution. This result demonstrates that the OECT‐sensor array can perform accurate and selective detections of multiple RNA sequence screening simultaneously and facilitate a fast and accurate disease diagnosis. According to the error bar presented in Figure 4d, the RNA concentration can only be determined with an accuracy of one order of magnitude. This OECT biosensor based on the interaction between RNA and conjugated polymers exhibits the lowest detection limit in comparison with other OECT‐based nucleic acid detection methods (Table S3).

## 3 Conclusion

In summary, we have studied the interaction between RNA and the organic semiconductor p(g2T‐TT) in aqueous solutions by measuring OECT performance. We observed for the first time that the volumetric capacitance of p(g2T‐TT) layer in an electrolyte decreased due to negatively charged RNA molecules. Specifically, the bulk doping of p(g2T‐TT) polymer by small anions in an aqueous solution is prohibited by the negatively charged biomolecules attached on the polymer chains due to electrostatic interactions. This interaction between conjugated polymers and biomolecules is expected to be observed in many other organic bioelectronic systems, which paves a way for the further development of organic bioelectronics with novel functions. Based on this effect, OECT arrays have been successfully used as label‐free RNA sensors with both high sensitivity and selectivity. Several RNA biomarkers for COVID‐19 are successfully detected simultaneously in aqueous solutions with a detection limit down to 10^−17^ M level, indicating that OECTs can be used as a multiplexing sensing platform for the diagnosis of diseases with low cost and fast speed.

## 4 Materials and Methods

### 4.1 Materials

Phosphate buffered saline (PBS) solution (pH = 7.4), chloroform, sodium chloride and sodium glycolate were purchased from Sigma‐Aldrich Co., USA. Sodium poly(styrene sulfonate) (Mw 75000, 30% w/v aqueous solution) was purchased from Alfa Aesar. Poly(vinyl alcohol) 1788 (87.0% ∼ 89.0% mol/mol) (PVA) was purchased from Macklin Inc. AZ5214 and SU‐8 2002 photoresists were purchased from Microchemicals GmbH. (3‐Glycidyloxypropyl) trimethoxysilane (GOPS) was purchased from International Laboratory, USA. Fluorescein isothiocyanate‐polyethylene glycol amine (FITC‐PEG‐NH_2_) was ordered from ShangHai ToYongBio Tech. Inc. Poly(2‐(3,3′‐bis(2‐(2‐(2‐methoxyethoxy)ethoxy)ethoxy)‐[2,2’‐bithiophen]‐5‐yl)thieno [3,2‐b]thiophene), p(g2T‐TT), was synthesized as previously reported. All the DNA and RNA sequences were ordered from Sangon Biotech Co., China.

### 4.2 Device Fabrication

OECT devices were fabricated in the following steps [38]. Briefly, a polyethylene terephthalate (PET) substrate was thoroughly cleaned, then Cr/Au (10/100 nm) electrodes were patterned on the PET substrate by photolithography and magnetron sputtering deposition. p(g2T‐TT) polymer and SU‐8 2002 insulating layers were then patterned in sequence, forming an active channel area with the channel width and length of 60 and 30 µm, respectively. The gate electrode area was defined to be 1000 µm × 600 µm after the lift off process, which is much bigger than the channel area. The p(g2T‐TT) polymer was dissolved in chloroform at 2 mg/mL, spin coated on the substrate and annealed at 100°C for 1 h before patterning.

To immobilize DNA probe molecules on the surface of OECTs, the p(g2T‐TT) channel area was first exposed to O_2_ plasma treatment (20 W/ 2 min) to generate hydroxyl functional groups, then GOPS was deposited by a vapor deposition method in a vacuumed desiccator at the temperature of 95°C for 1 h. Subsequently, a 1 µM amino modified DNA probe solution (adjusted to pH 9) was dropped on the channel for probe immobilization, through the ring‐opening reaction of the epoxy from GOPS with the amino group from DNA.

### 4.3 Device Characterization

A small droplet (10 µL) of an aqueous solution (0.1X PBS or other ionic electrolytes) was coated on the OECT to connect the channel area with the gate electrode. The output and transfer characteristics of the OECTs were measured by using two Keithley 2400 sourcemeters with a customized LabVIEW program. Electrochemical impedance spectroscopy (EIS) measurements of p(g2T‐TT) polymer films were performed with the Zahner Zennium pro electrochemical workstation and a three‐electrode‐system, including Indium tin oxide (ITO) electrodes (0.2 cm^2^) coated with thin film of p(g2T‐TT) polymer (working electrode), a platinum wire as counter electrode and an Ag/AgCl reference electrode. Effective capacitance was derived from 1/(2π*f*·Z_im_) and used for the calculation of areal and volumetric capacitance, where *f* is the frequency and Z_im_ is the imaginary part of the impedance.

Atomic Force microscopy (AFM) characterization was performed with a Bruker Nanoscope 8 microscope in tapping‐in‐air mode. The film thickness was recorded by Bruker Dektak XT surface profilometer. Fourier transform infrared spectroscopy (FT‐IR) was introduced for the direct characterization of polymer film surfaces using the attenuated total reflectance (ATR) accessory of Bruker Vertex 70 spectrometer.

### 4.4 Cell Culture and Treatment

Cell lines, A549 cell and Hela cell, are used for miRNA extraction procedure. All reagents used in the cell culture experiment are purchased from Thermo Fisher unless specified otherwise. Both cell lines are obtained from ATCC. A549 cell and Hela cell are cultured in Ham's F‐12K (Kaighn's) Medium and Dulbecco's Modified Eagle Medium at 37°C in a humidified atmosphere of 5% CO_2_, respectively, both of which are supplemented with 10% fetal bovine serum and 1% penicillin/streptomycin solution. During cell culture, the medium is changed every other day. The culture is split when it reaches 80% confluence. When the sub‐cultured cells enter the log phase, they are trysinized and thoroughly washed with PBS solution. Then washed cell pellets are transfer to RNase‐free tubes for further miRNA extraction process. The extractions of miRNA from both cancer cells are performed using PureLink miRNA isolation Kit from Thermo Fisher (Catlog Number: 157001). The detail extraction process can be found in the online user guide of the Kit. The extracted miRNA is dissolved in RNase water and the total extracted miRNA in each sample can be determined by NanoDropTM spectrophotometers. In order to avoid miRNA degradation, the extracted miRNA samples are immediately stored in −80°C after isolation for further use.

### 4.5 Statistical Analysis

In this work, normalized responses are employed to mitigate slight variations of initial currents of different OECT devices. The normalized current response is defined as: *ΔI/I* = (*I*_DNA_−*I*_RNA_)/*I*_DNA_, where *I*_DNA_ is the current measured at *V_G_* = –0.6 V after DNA probe modification, and *I*_RNA_ is the current measured at the same gate voltage after RNA interaction. The normalized capacitive response is defined as: *ΔC/C*, where *C* is capacitance of p(g2T‐TT) films after DNA probe modification, *ΔC* is the change of capacitance after RNA interaction. In this work (Figures 2, 3, 4), the normalized responses corresponding to varying RNA concentrations are presented as mean ± standard deviation (SD). Error bars denote the SD derived from at least three independent replicates (n = 3).

Dose‐response curves were fitted to the device responses obtained at varying RNA concentrations (Figures 3 and 4). The data were fitted using the following sigmoidal function:

$$Y=\frac{A_{1}-A_{2}}{1+{\left(\frac{X}{X_{0}}\right)}^{P}}+A_{2}$$

where *X* is the concentration of RNA, *Y* is the normalized current response, *A_1_* represents the approximate estimate of the upper asymptote, *A_2_* represents the approximate estimate of the lower asymptote of the curve, *P* is the slope of the curve, and *X_0_* is the concentration corresponding to half of the maximum response value. All data statistical analysis is completed through Origin software.

## Author Contributions

F.Y. conceived the concept and supervised the work. H.L. and N.W. fabricated devices, conducted the experiments, and prepared the figures. I.M. provided the polymer p(g2T‐TT). Y.F., A.Y., S.J., H.K.L., and L.L. assisted on the experiments. F.Y., N.W. and H.L. wrote the manuscript. All authors were involved in the preparation and discussion of the manuscript.

## Conflicts of Interest

The authors declare no conflicts of interest.

## Supporting information




**Supporting File**: adma73802‐sup‐0001‐SuppMat.docx.

## Data Availability

The data that support the findings of this study are available from the corresponding author upon reasonable request.
