# Supplementary material for: Intimate Interaction Between Nucleic Acid and Conjugated Polymers in Organic Electrochemical Transistors Enables Ultrasensitive Biomarker Detection
Source: Adv Mater. 2026 Jun 25;38(43):e73802. doi: 10.1002/adma.73802 (PMC13432185; doi:10.1002/adma.73802)
Supplement: Supplementary file 1 — Supporting File: adma73802‐sup‐0001‐SuppMat.docx. [file ADMA-38-e73802-s001.docx]

Supporting Information

Intimate Interaction between Nucleic Acid and Conjugated Polymers in Organic Electrochemical Transistors Enables Ultrasensitive Biomarker Detection

Hong Liu^1^, Naixiang Wang^1^, Anneng Yang,^1^ Jiajun Song,^1^ Li Li,^2^ Iain McCulloch,^3,4^ Helen Ka-wai Law,^5^ Feng Yan*^1,6^

*E-mail: apafyan@polyu.edu.hk*

**Table of contents:**

Supplementary Notes S1–S3

Supplementary Figures S1–S21

Supplementary Tables S1–S3

References


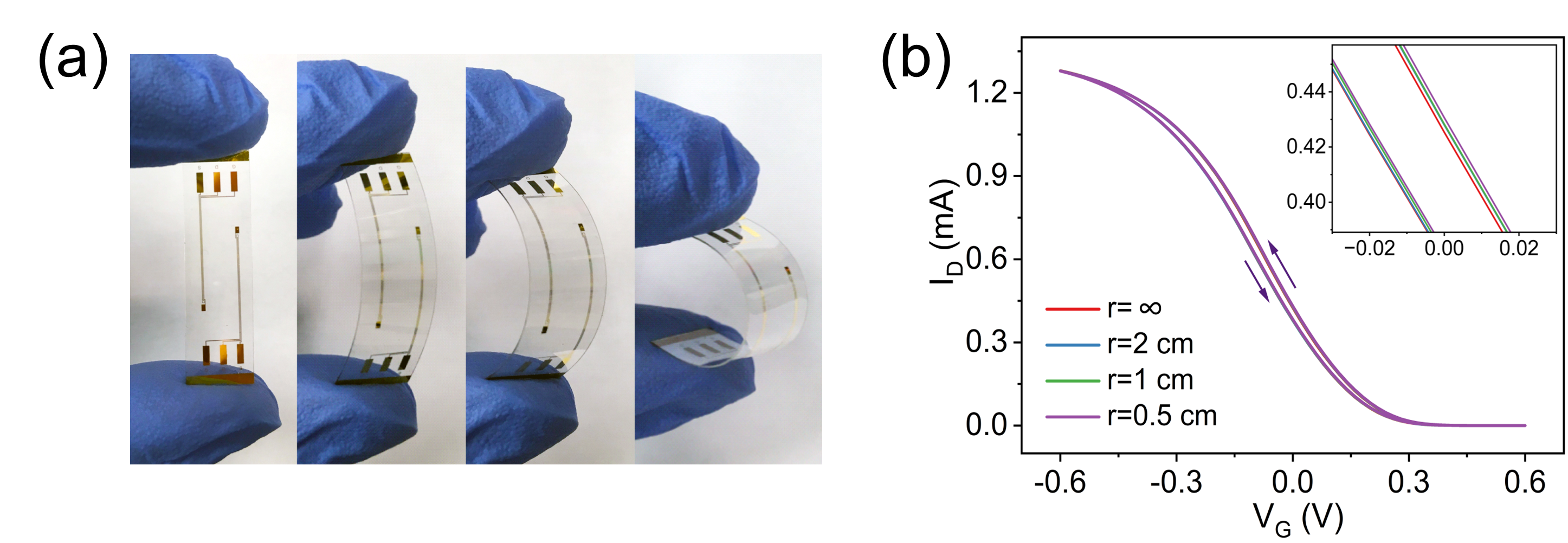


Figure S1│Performance of flexible OECT devices. (a) Photographs of a flexible OECT with different bending statues; (b) Transfer characteristics (drain current I_D_ versus gate voltage V_G_) of an OECT with different bending radii.


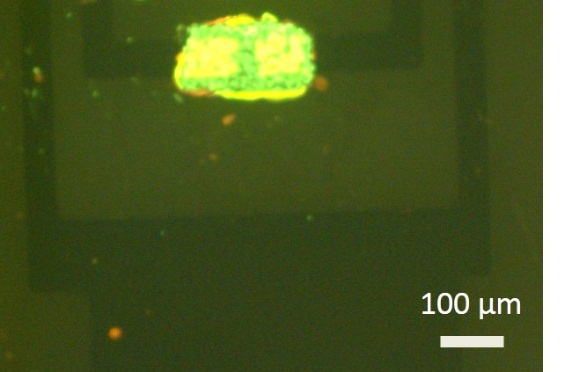


**Figure S2│Fluorescence microscopy image of amino fluorescein immobilized on the surface of GOPS -modified p(g2T-TT) channel.**


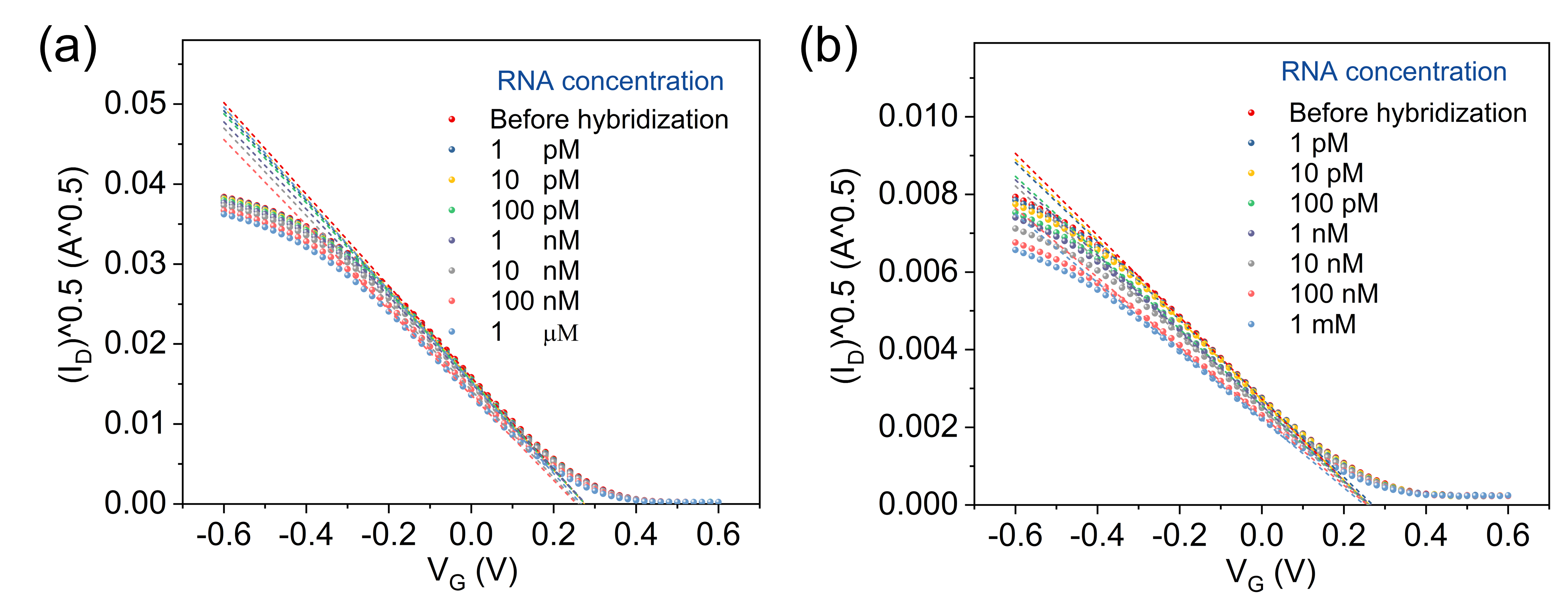


**Figure S3│Determination of threshold voltages of OECTs after RNA hybridization.** Square root of drain current I_D_ as a function of V_G_ for OECTs with channel thicknesses of (a) 100 nm, (b) 20 nm. Tangents are drawn for each transfer curve for determination of threshold voltage. V_DS_ = − 0.5 V.


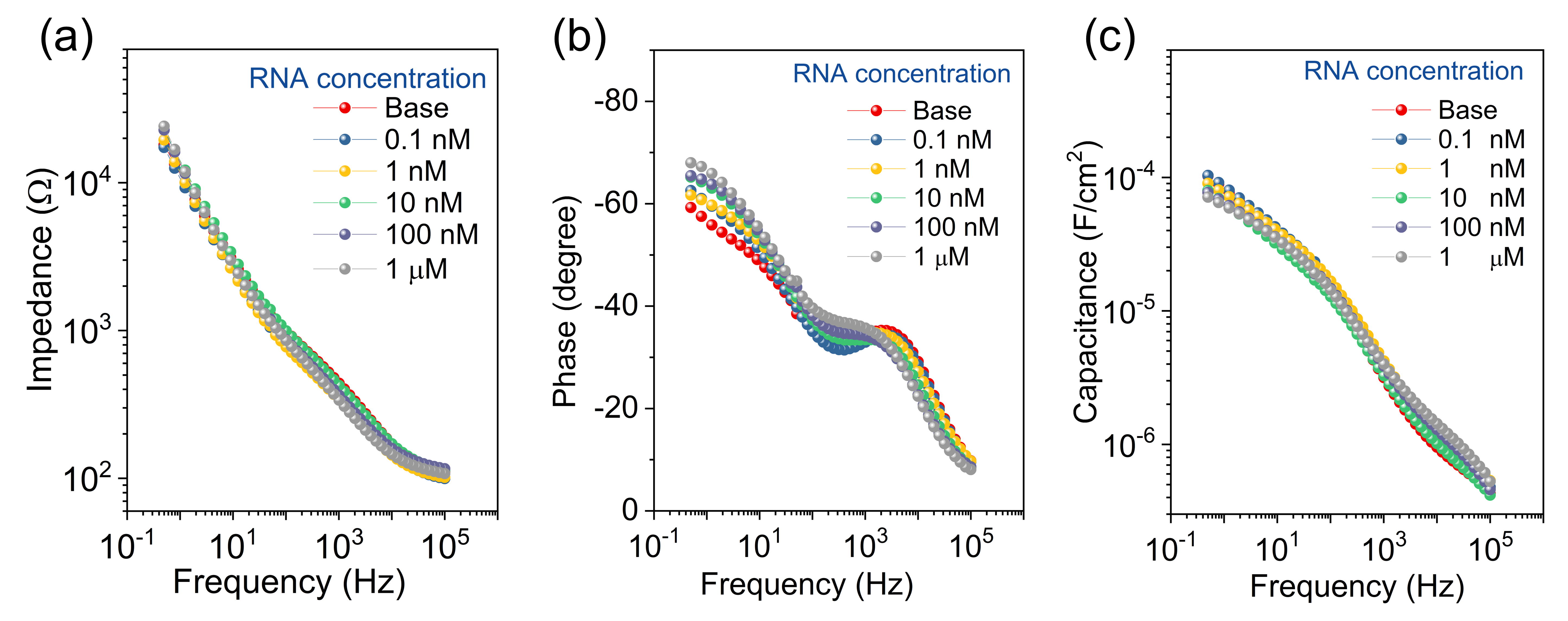


**Figure S4│ Electrochemical impedance spectroscopy (EIS) measurements on p(g2T-TT) films.** (a) Impedance, (b) phase angle, and (c) effective areal capacitance versus frequency, from EIS for 20 nm thick p(g2T-TT) film coated on ITO substrate and modified with increasing complementary RNA concentrations.


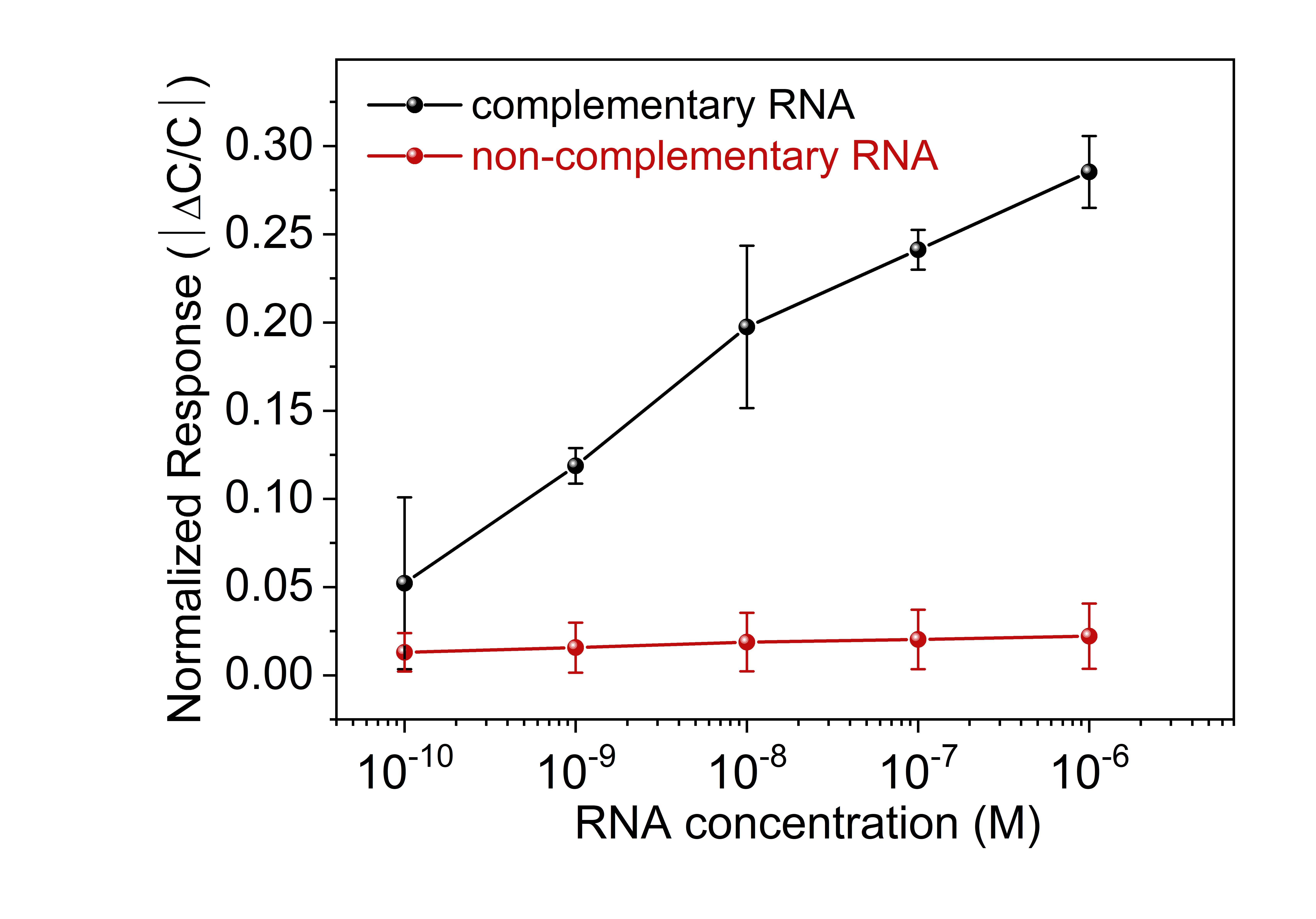


**Figure S5│Normalized capacitance versus the concentrations of complementary and non-complementary RNA molecules.** The capacitance was measured at a frequency of 0.5 Hz. The thickness of p(g2T-TT) films is 20 nm.


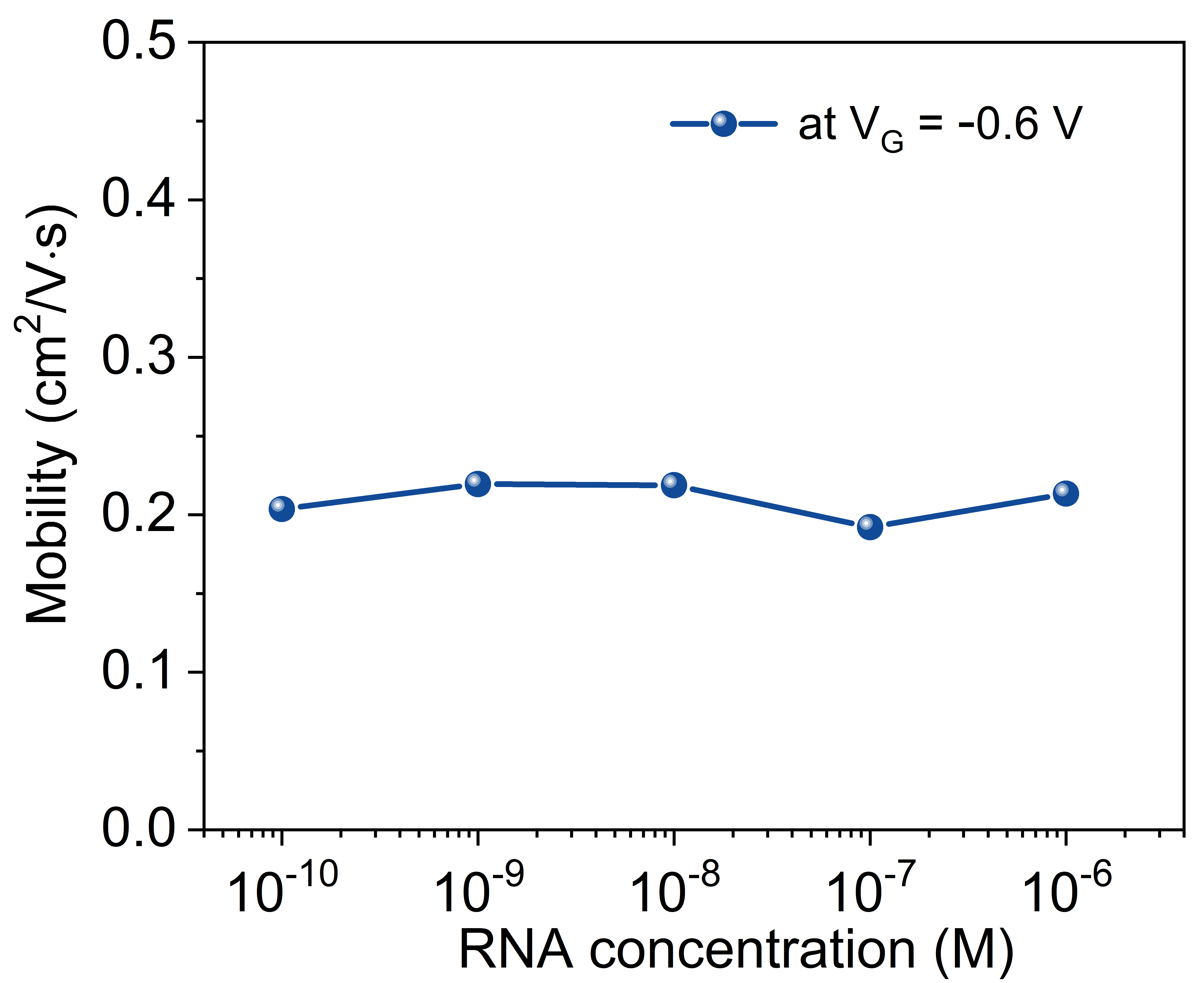


**Figure S6│** **Extracted carrier mobility with increasing RNA concentrations.** Carrier mobilities calculated from the saturation region of transfer characteristics (V_G_ = − 0.6V) under increasing RNA concentrations.


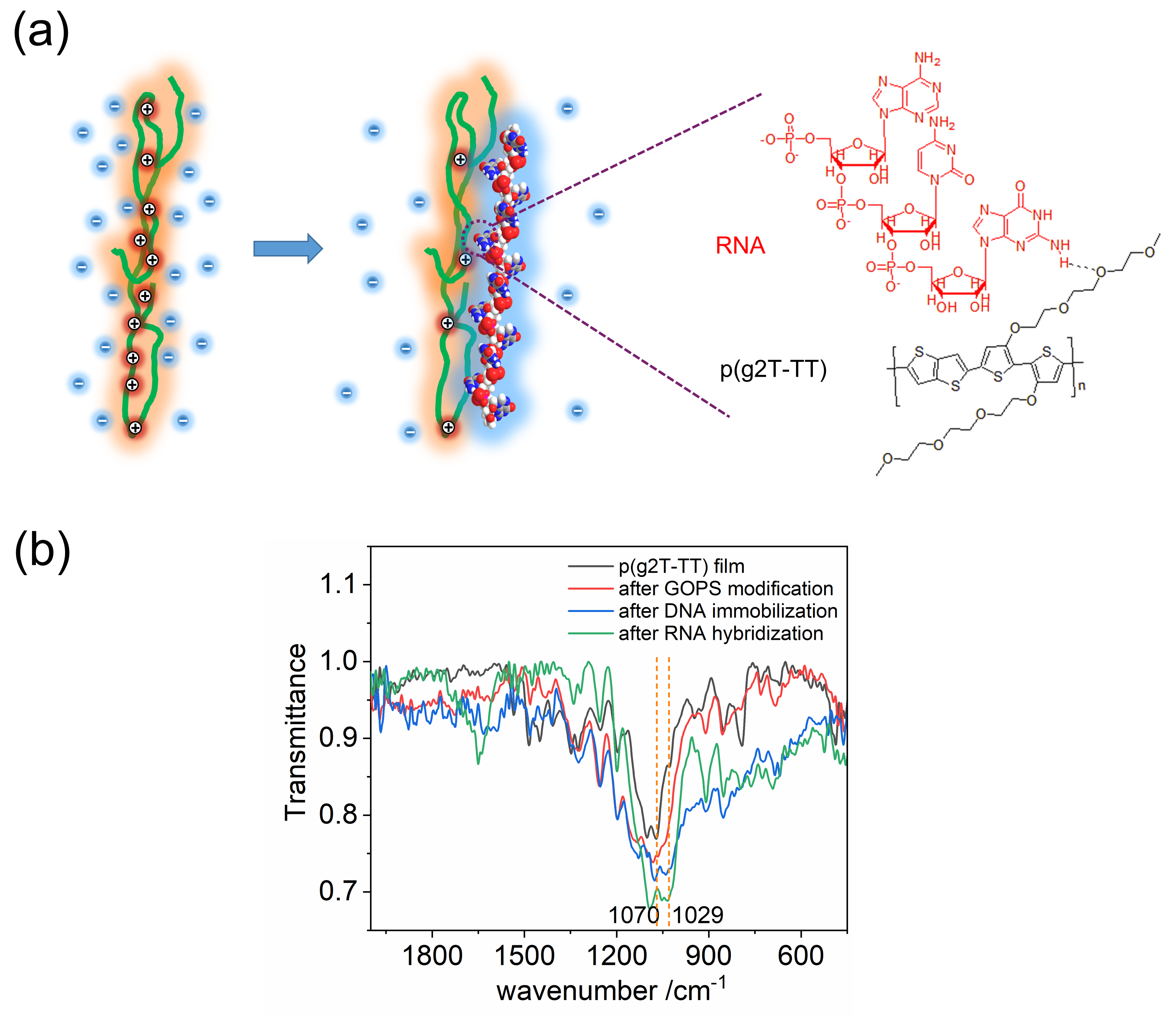


**Figure S7│Interactions between RNA molecules and p(g2T-TT) chains.** (a) Schematic diagram of the electrochemical doping process on p(g2T-TT) chains (green line) without (left) and with (right) the existence of RNA molecules (space-filling model); (b) FT-IR spectra of p(g2T-TT) films (black), after GOPS modification (red), after DNA immobilization (blue) and after the hybridization of RNA (green).

**Note S1|** **A cylinder model for the interaction between p(g2T-TT) and RNA**

When the distance *s* between p(g2T-TT) and RNA helix is small enough, the overlap on the electrical double layer (EDL) shell will lead to a redistribution of ions around the molecules and affect the capacitance of the polymer. As shown in supplementary Figure 7a, when an overlap exists between the EDLs of p(g2T-TT) and RNA helix, the surface area of the EDL around the p(g2T-TT) polymer chain is decreased, leading to decreased volumetric capacitance. Thus, the capacitance *C* of p(g2T-TT) is approximately given by:

$C\approx C_{0}\cdot\left( 1-\frac{2*(2\pi-\alpha)}{2\pi} \right)$ ,

where, *C_0_* is the intrinsic capacitance of (g2T-TT) without the effect of RNA helix and α is the overlap angel of the two cylinders. Assuming the distance between the two cylinders is s, the overlap angle is given by:

$\alpha=2\cos^{-1} \frac{r_{1}^{2}+s^{2}-r_{2}^{2}}{2r_{1}s}$,

where *r_1_* and *r_2_* represent the radius of the cylinder model of p(g2T-TT) and RNA helix separately. According to previous investigations, the diameter of DNA/RNA helix is approximately 2.6 nm,^[1]^ and the EDL thickness is around 2.4 nm in 0.1X PBS solution.^[2]^ For p(g2T-TT) polymer chain, it is assumed that the radius depends on the length of the glycolated side chain, which is estimated as 1.49 nm^[3]^. Therefore, we get the parameters *r_1_* = 1.49 + 2.4 = 3.89 nm, and *r_2_* = 2.6/2 + 2.4 = 3.7 nm. The relationship between *C/C_0_* and distance *s* could then be calculated and plotted out. As shown in supplementary Figure 7b, when the distance is reduced to 4.1 nm, a 30 % capacitance decrease is observed for p(g2T-TT), which is consistent with the experimental results from OECT sensors and p(g2T-TT) films.


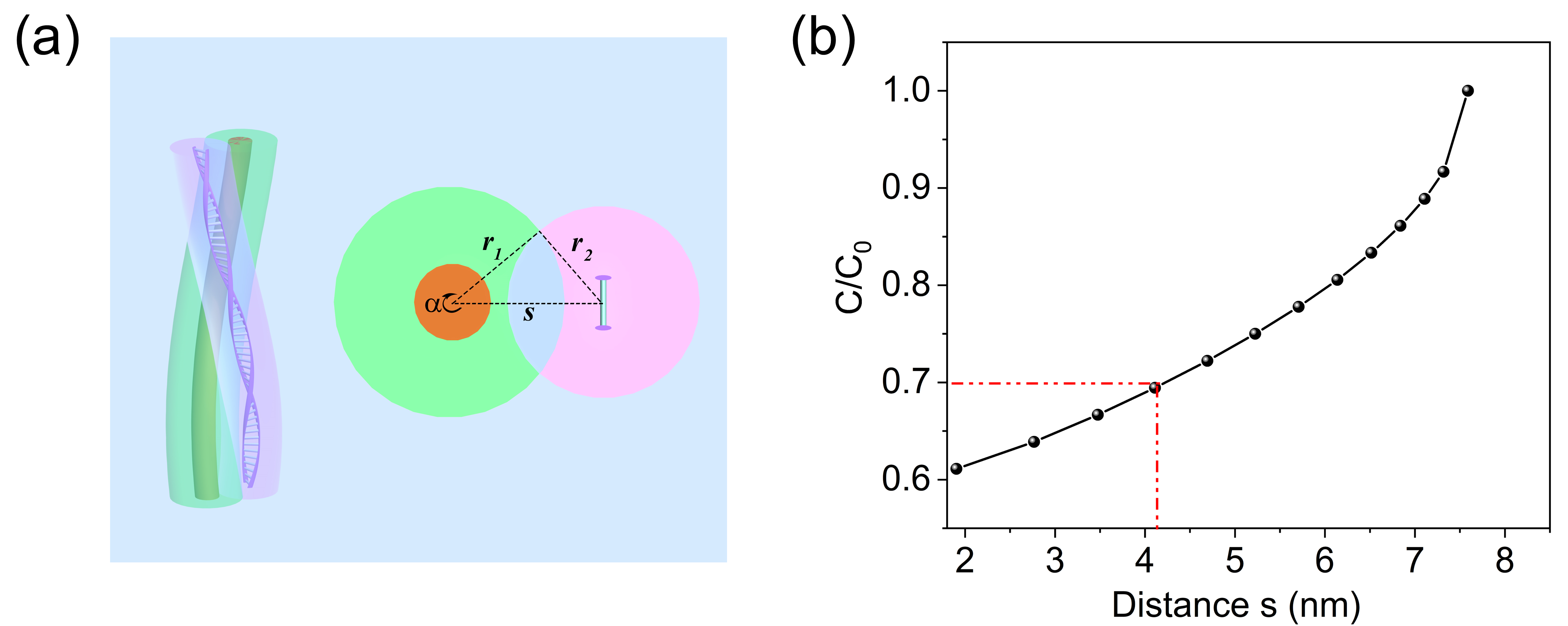


**Figure S8│The influence of an RNA molecule on the surface capacitance of a p(g2T-TT) polymer chain in an electrolyte.** (a) A cylinder model to mimic the effect of RNA helix on the capacitance of p(g2T-TT) polymer. The green and pink cylinders represent the electrical double layer of p(g2T-TT) and RNA helix separately. s is the distance between p(g2T-TT) and RNA helix. (b) Calculated capacitances decrease of p(g2T-TT) versus the distance s, according to the cylinder model.


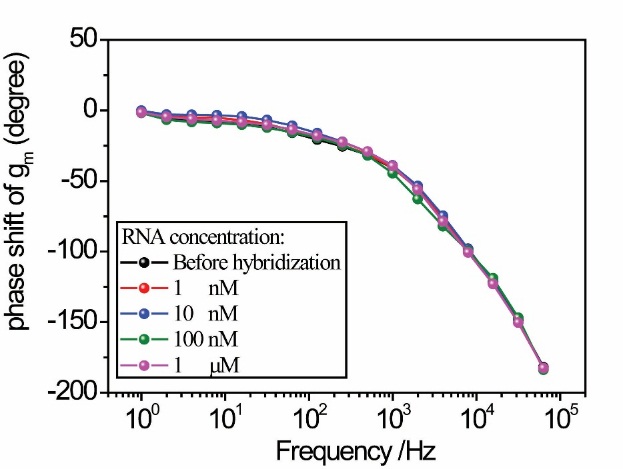


**Figure S9│Transconductance spectrum characterization.** The phase angle shift of transconductance (g_m_) versus frequency of DNA probe modified OECT upon addition on increasing concentration of miRNA-21.


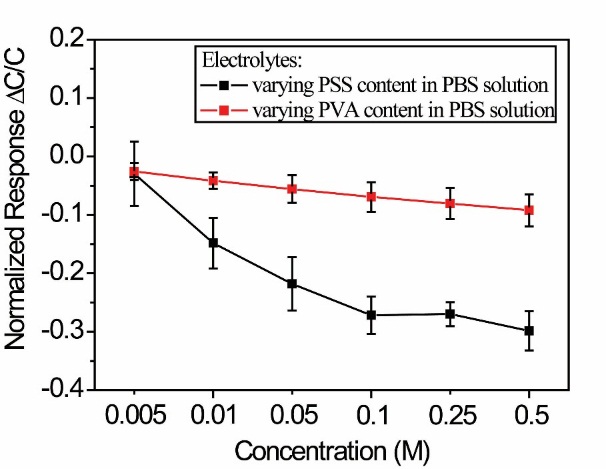


**Figure S10│Capacitance change with PSS or PVA in electrolytes.** Normalized capacitance changes of p(g2T-TT) film (thickness: 20 nm) were characterized in PBS solution with varying PSS (black) or PVA concentrations (red) measured at a frequency of 0.5Hz (V_bias_ = 0.6 V). The concentration is calculated based on repeat units, representing the concentration of negative charges in PSS or hydroxyl groups in PVA.

**Note S2| DNA/RNA and polymer structure modeling**

The AlphaFold 3 model was employed to accurately predict the interactions between proteins, nucleic acids, and small molecules. The structure of the DNA, RNA and polymer were constructed using Chem3D, as shown in supplementary Figure 10 a-c. Subsequently, the molecular structure was optimized, and PM3 atomic charges were calculated using the MOPAC program for subsequent molecular docking. The ligand was prepared with AutoDock Tools 1.5.6 to generate the PDBQT file. Docking simulations were performed with AutoDock 4.2.6, using a cubic box (approx. 50 Å × 50 Å × 50 Å) centered on the DNA to encompass the entire structure, in an aqueous solution containing Na⁺ and Cl⁻ ions. The results showed a binding energy of – 4.534 kcal/mol with an DNA-RNA hybrid, suggesting a stable polymer-DNA-RNA complex.

The polymer binds stably to the DNA-RNA hybrid through the synergistic action of both hydrogen bonding and hydrophobic interactions. Supplementary Figure 10d depicts the two-dimensional interaction diagram between the polymer and the DNA-RNA hybrid. Supplementary Figure 10e presents the three-dimensional view of their hydrogen-bonding network. As shown, the polymer molecule docks stably into cavities formed by specific bases: dt21, da22, dg10, dc12, dt1, dt11, and da9 from the DNA strand, and g3, u6, u5, c4, a2, u14, u17, a16, g15, and c13 from the RNA strand. Interaction analysis reveals the formation of hydrogen bonds between the polymer and base dg10 (DNA strand) and base g15 (RNA strand) within the cavity. These interactions effectively exclude water molecules, thereby enhancing the stability of the complex.


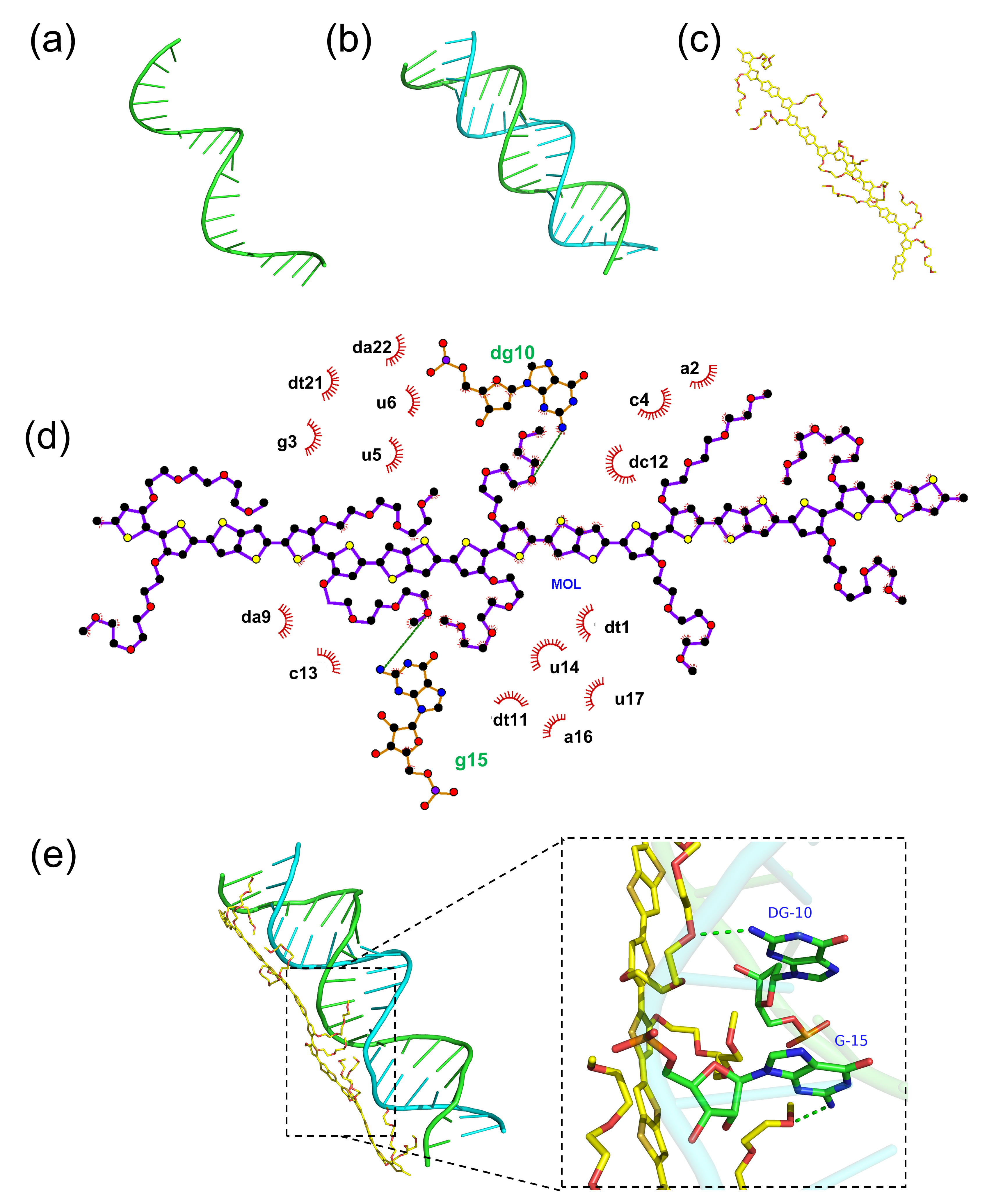


**Figure S11│Structures of DNA, RNA and p(g2T-TT) polymer and molecular bindings.** (a) a DNA strand. (b) DNA-RNA hybrid. (c) p(g2T-TT) polymer. (d) Two-dimensional binding mode diagram of the polymer molecule with the DNA-RNA hybrid. Hydrophobic interactions are indicated by red gear-shaped symbols, and hydrogen bonds are represented by green dashed lines. (e) Position of the polymer within the three-dimensional structure of the DNA-RNA hybrid (left); Detailed 3D binding mode illustration of the polymer with the DNA-RNA hybrid (right).

**Note S3| Molecular dynamics simulations**

The molecular dynamics simulations were performed using Gromacs 2022.1 with the Amber14SB force field. The system was solvated in a cubic box of 8.0 nm × 8.0 nm × 8.0 nm under periodic boundary conditions. All interactions were described using the all-atom Amber14SB force field. Energy minimization of the initial structure was conducted for 50,000 steps using the steepest descent algorithm. The simulations were carried out in the NPT ensemble. The equations of motion were integrated using the leap-frog algorithm. The Particle Mesh Ewald (PME) method was employed for handling long-range electrostatic interactions. The cutoff radii for van der Waals and Coulomb interactions were both set to 12 Å, with the neighbor list updated every 10 steps. All bonds were constrained using the LINCS algorithm with the parameters linkster = 1 and lincsorder = 4. The system temperature gradually increased from 0 K to 298.15 K using the V-rescale thermostat. Pressure was maintained at 1 bar using the Parrinello-Rahman barostat, with isotropic pressure coupling. A neighbor-search grid scheme was utilized for non-bonded interaction calculations, with short-range and long-range cutoff distances set at 9 Å and 14 Å, respectively. A hydrogen bond was defined based on a donor-acceptor distance of less than 0.35 nm and a donor-hydrogen-acceptor angle of less than 30°. Initial atomic velocities were assigned randomly according to a Maxwell-Boltzmann distribution at the target temperature. The production simulation was run for 50 million steps with a time step of 2 fs, resulting in a total simulation time of 100 ns. Throughout the trajectory, 10,000 frames were saved for analysis. Visualization and analysis of the trajectories were performed using the built-in tools of Gromacs and the VMD software.

Following the introduction of RNA, the system exhibits series of changes, as presented in supplementary Figure 11. (1) First, the average centroid distance between the polymer and the nucleic acid decreases from 1.815 nm to 1.68 nm. (2) The radius of gyration (Rg) of the system decreases from 2.313 ± 0.232 nm to 2.051 ± 0.052 nm over the simulation trajectory (supplementary Figure 11a), with the smaller value and reduced fluctuations. The radius of gyration (Rg) serves as a key metric for assessing the overall compactness of DNA structures. Larger fluctuations in Rg indicate greater conformational changes within the system. The smaller fluctuation indicates a more stable conformation of the DNA-RNA-polymer complex after hybridization. (3) The solvent-accessible surface area (SASA) characterizes the surface topography and effective volume of the polymer; a compaction of the structure leads to a reduction in the SASA value. As presented in supplementary Figure 11b, the SASA of the polymer with DNA-RNA remained relatively stable during the simulation. After the introduction of RNA, the average SASA decreased from 39.086 ± 1.926 nm² to 36.628 ± 1.551 nm². The observed reduction and stabilization of the SASA suggest enhanced intramolecular interactions within the system following RNA incorporation. (4) Moreover, the average number of hydrogen bonds in the polymer-DNA complex increased from 0.621 to 0.827 following RNA hybridization (supplementary Figure 11c). The increase in hydrogen bond count further confirms the stronger interactions within the polymer-DNA complex facilitated by RNA hybridization. (5) The binding free energy changes from – 241.244 kJ/mol to – 285.347 kJ/mol (supplementary Table 1), consistent with stronger overall interactions in the system.


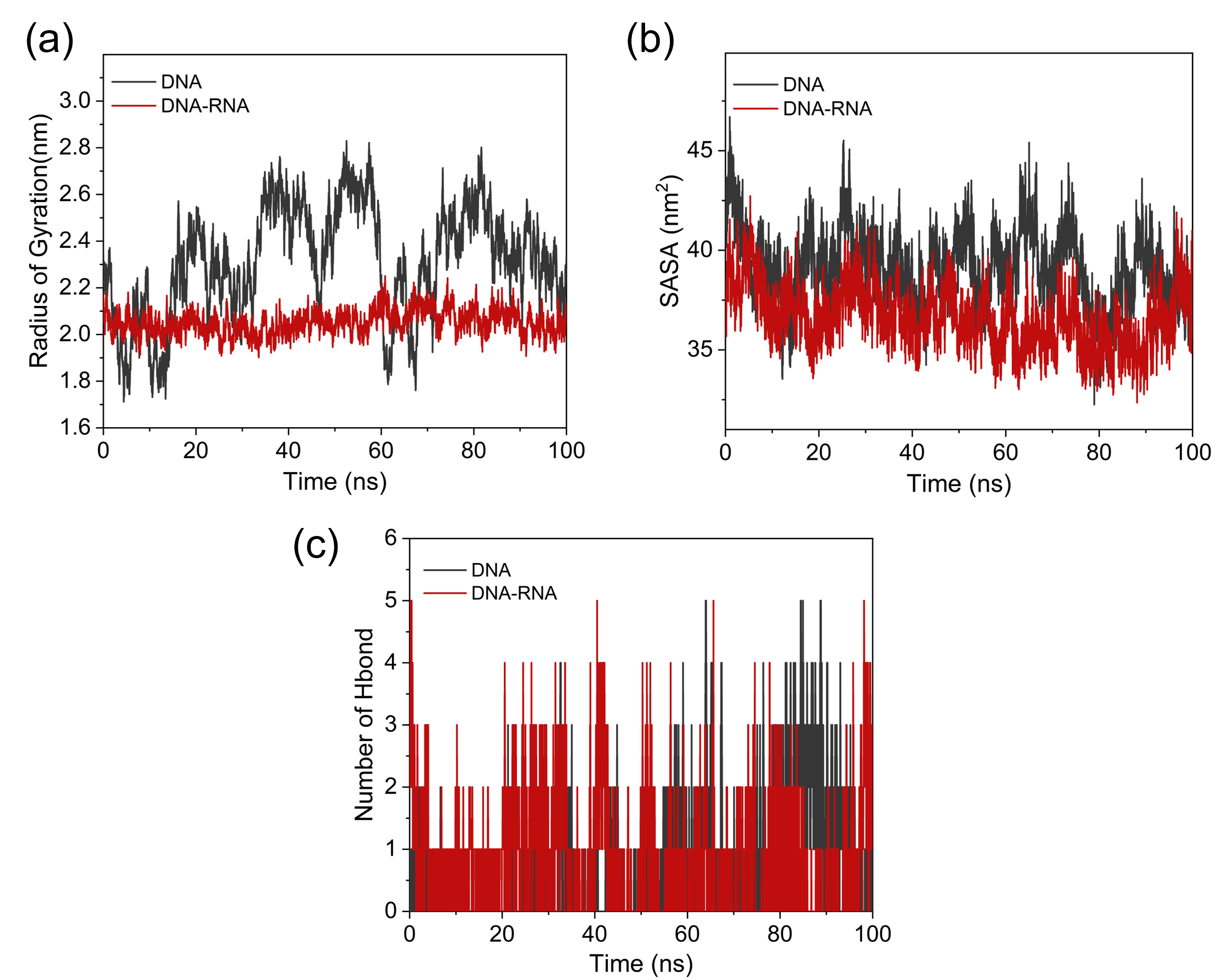


**Figure S12│** **Atomistic molecular dynamics simulation.** (a) Radius of gyration as a function of simulation time; (b) Solvent-accessible surface area (SASA) as a function of simulation time; (c) Number of hydrogen bonds between the polymer molecule and the DNA–RNA hybrid as a function of simulation time.


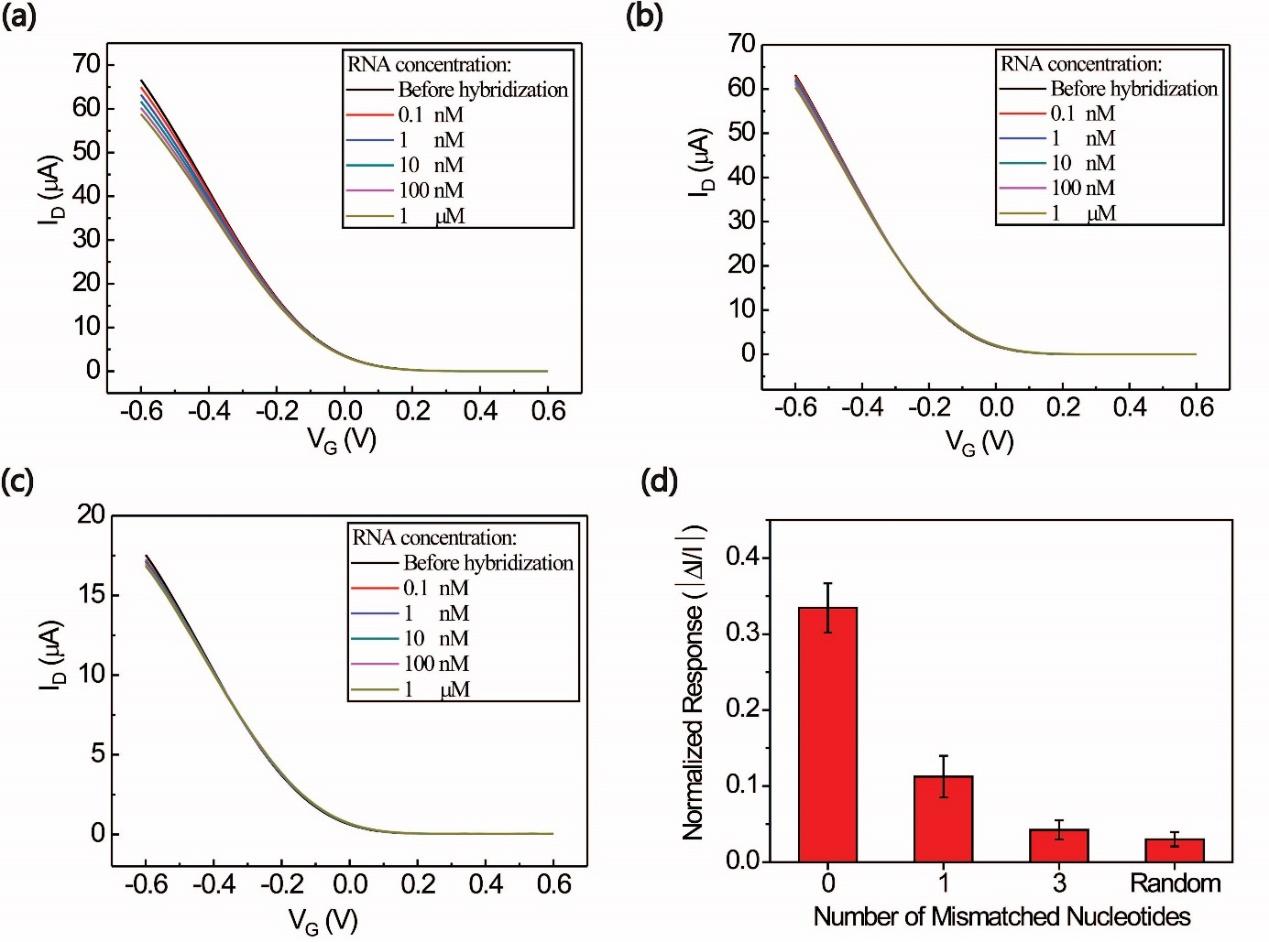


**Figure S13│Selectivity tests of the OECT-based RNA sensors.** The change in transfer curves of the OECT based RNA sensor when exposed to (a) single-base mismatched RNA target, (b) three-base mismatched RNA target and (c) non-complementary RNA target with random sequence. (d) Normalized current decreases of the OECT-based sensors for the detections of complementary RNA target, single-base mismatched, three-base mismatched and non-complementary RNA molecules. V_DS_ = − 0.5 V. Error bar was calculated based on the responses of at least 3 identical devices.


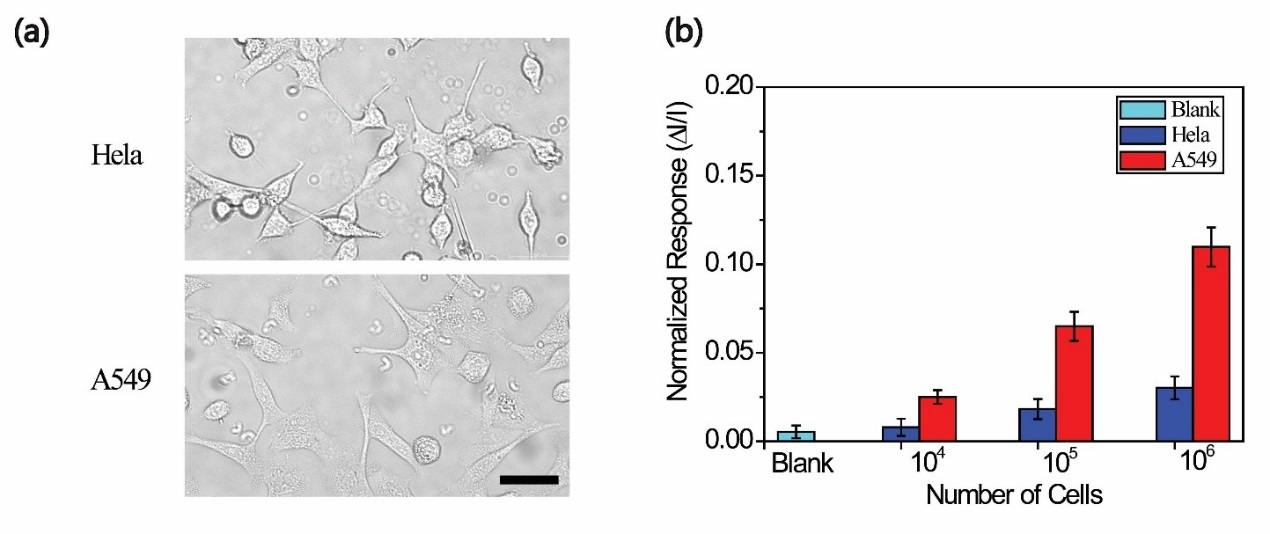


**Figure S14│Detection of a miRNA-21 biomarker from cancer cell lines.** (a) Optical images of Hela and A549 cancer cell lines in culture media. Scale Bar: 100 µm. (b) Normalized response of the OECT-based sensors for the detections of target miRNA-21 from the blank condition, Hela and A549 cancer cell lines with different cell numbers.


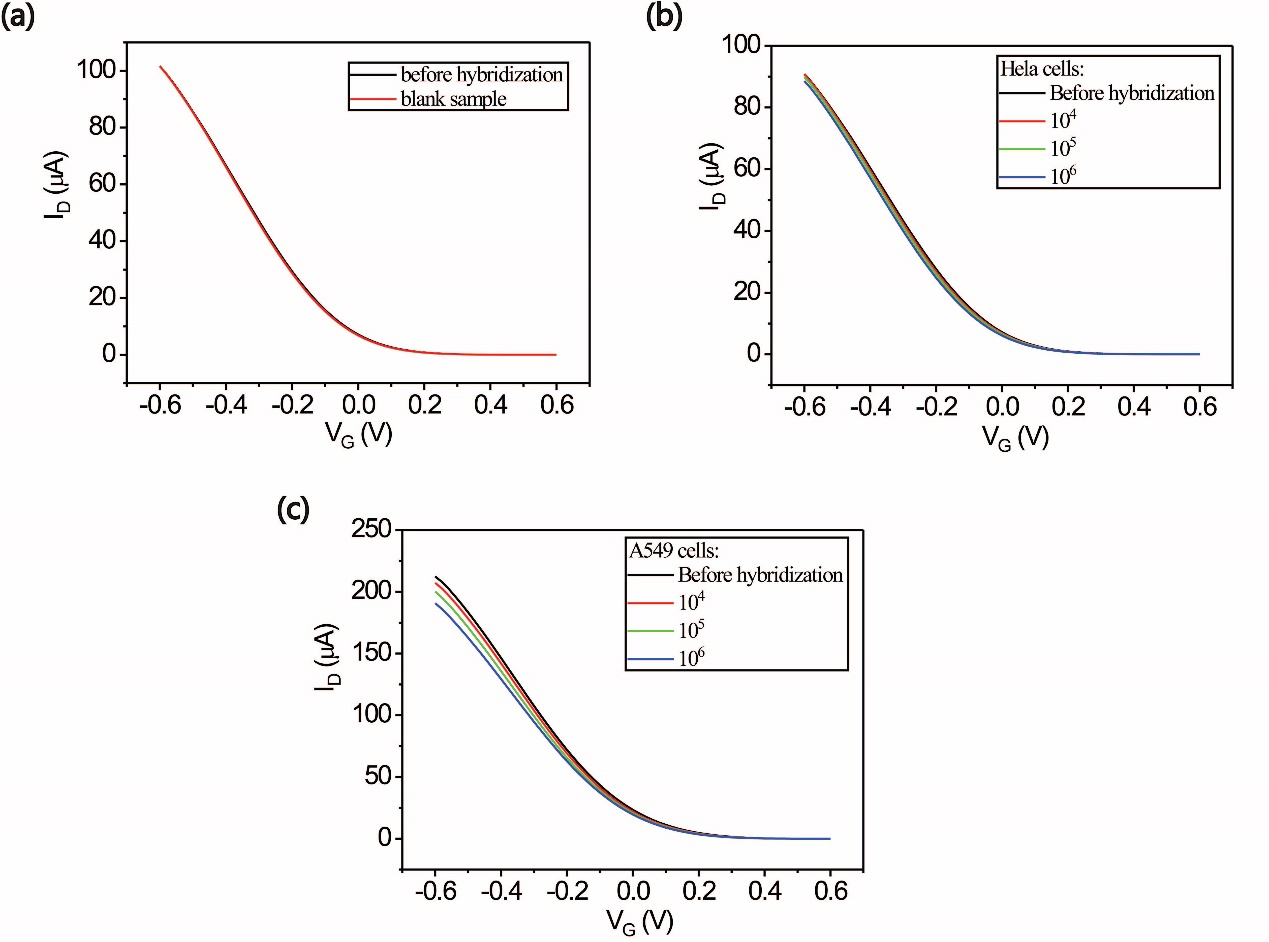


**Figure S15│Sensor response to different cancer cell lines.** The change in transfer curves of the OECT-based RNA sensor when exposed to (a) blank sample, (b) Hela cell and (c) A549 cell culture extracts with increasing cell numbers from 10^4^ to 10^6^. V_DS_ = − 0.5 V.


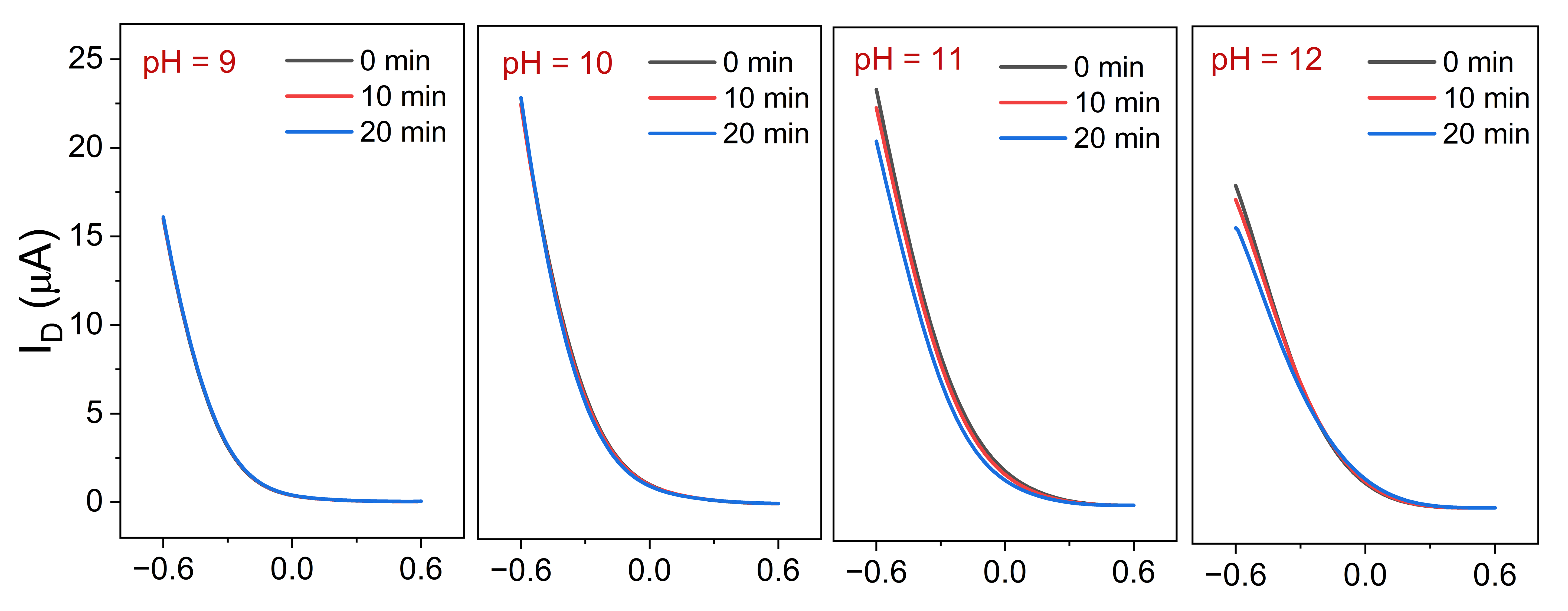


**Figure S16│**The stability of p(g2T-TT) device in electrolyte (0.01X PBS) with different pH values. The testing interval for each transfer curve was 10 minutes.


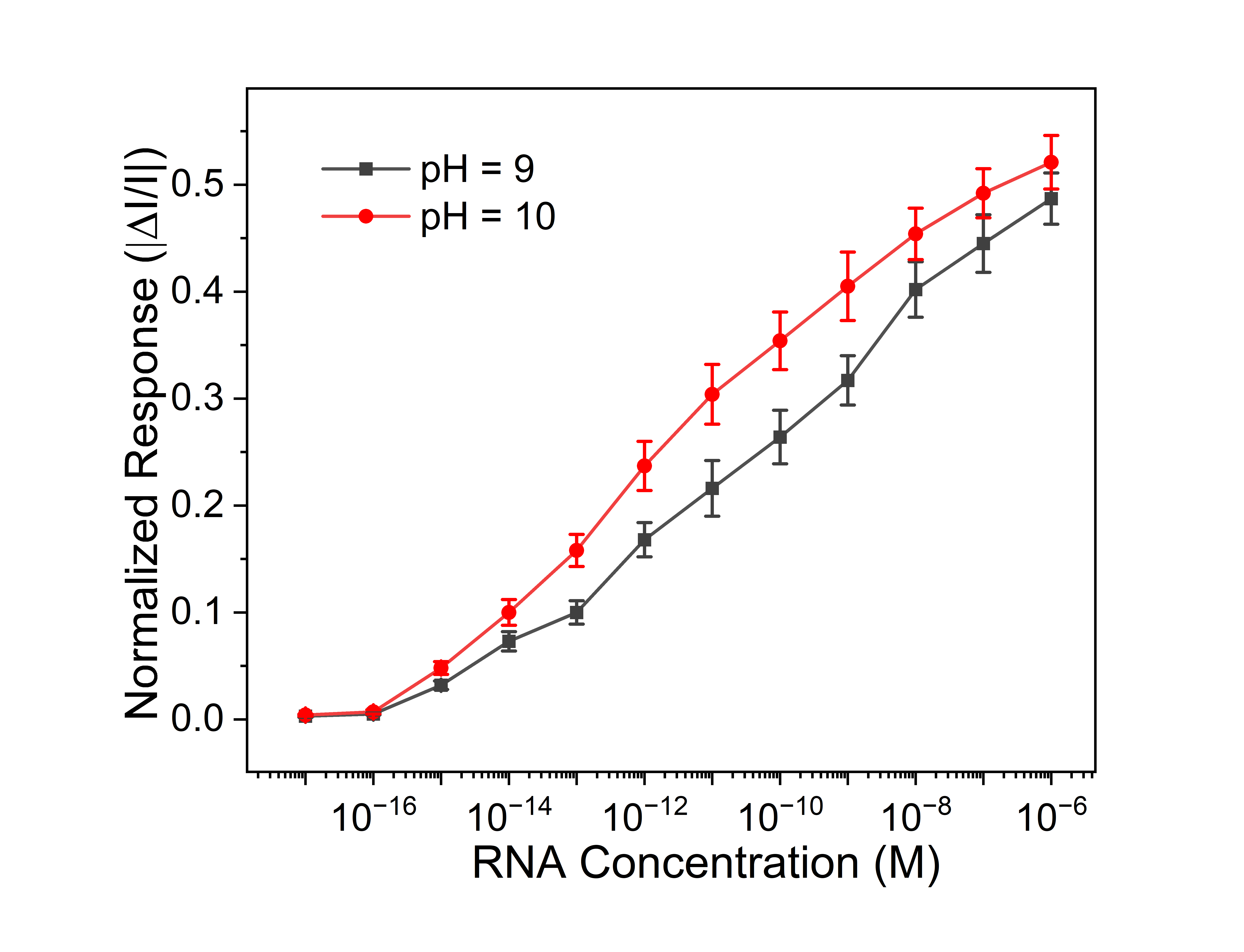


**Figure S17│Normalized current responses of the OECT devices to RNA.** The OECT devices were characterized in electrolytes with different pH values.


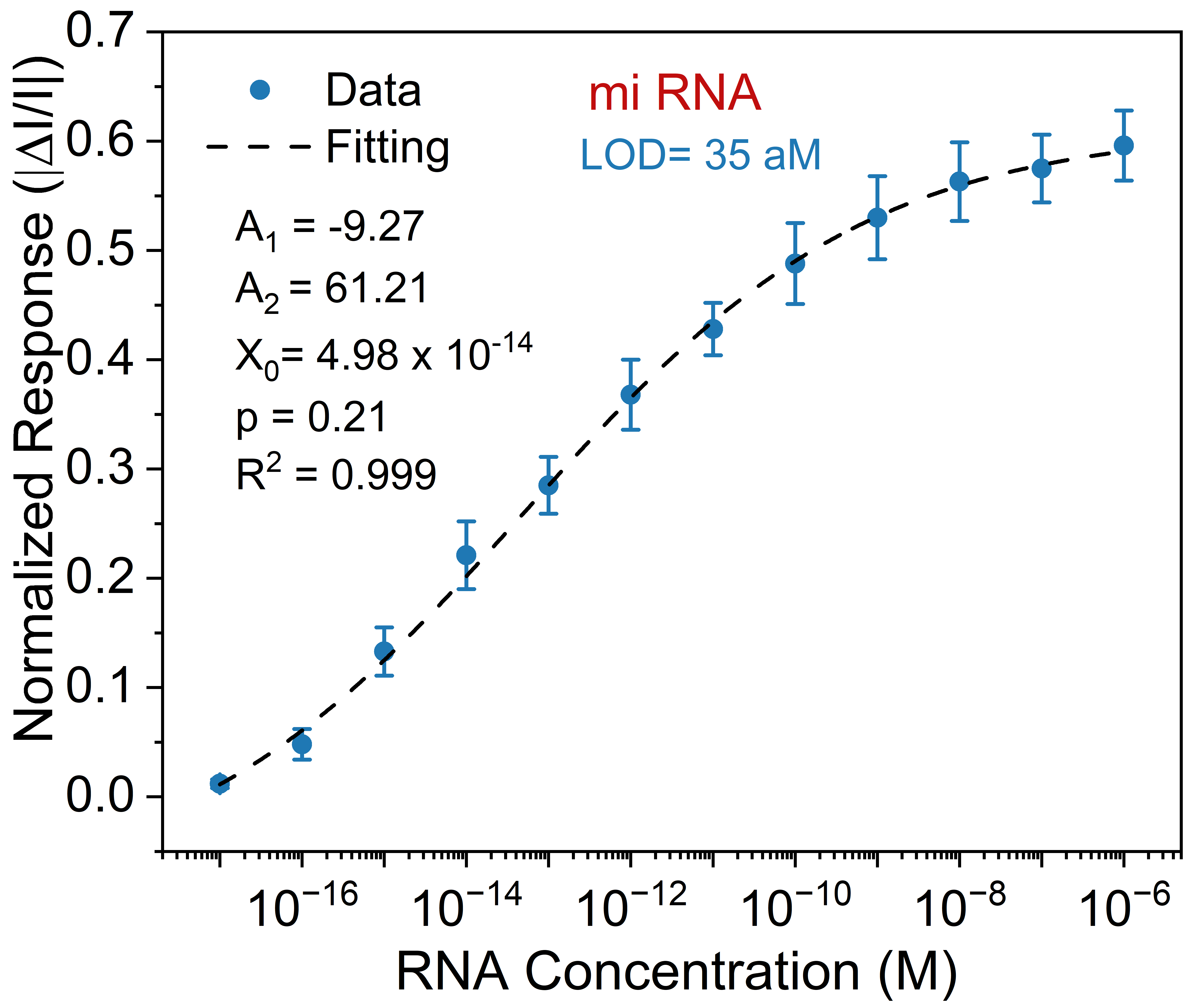


**Figure S18│Dose-response fitting for normalized current as a function of analyte concentration.** R^2^ = 0.999, and detection limit of mi RNA is 35 aM.

To simulate dose-response curves for test results, a sigmoidal (S - shaped) function is employed. The formula is as follows:

$$Y=\frac{A_{1}-A_{2}}{1+\left( \frac{X}{X_{0}} \right)^{P}}+A_{2}$$

In this formula, *X* is the concentration of the test substance, *Y* is the normalized response value of the current, *A_1_* represents the approximate estimate of the upper asymptote, *A_2_* represents the approximate estimate of the lower asymptote of the curve, *P* is the slope of the curve, and *X_0_* is the dose corresponding to half of the maximum response value. The R² value is 0.999, indicating excellent goodness of fit. Based on the fitted curve and the background signal level, the limit of detection (LOD) was determined to be 35 aM.


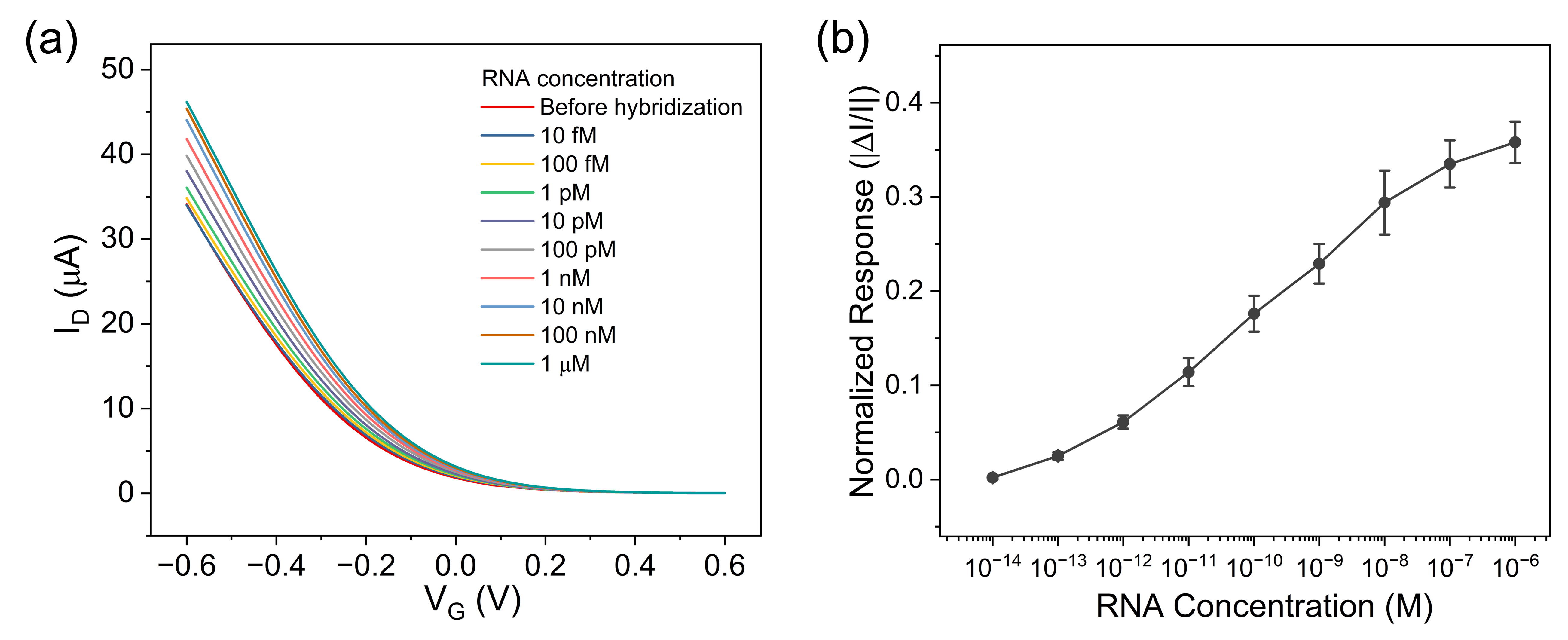


**Figure S19│OECT sensing response to RNA with gate as the biorecognition site.** (a) The change of transfer curves after reacting with RNA at different concentrations, (b) normalized current response as the increase of RNA concentration. The test electrolyte is 0.01X PBS with pH value of 10. A voltage pulse (−0.5 V, 1 kHz, 10 min) was applied during the hybridization process.


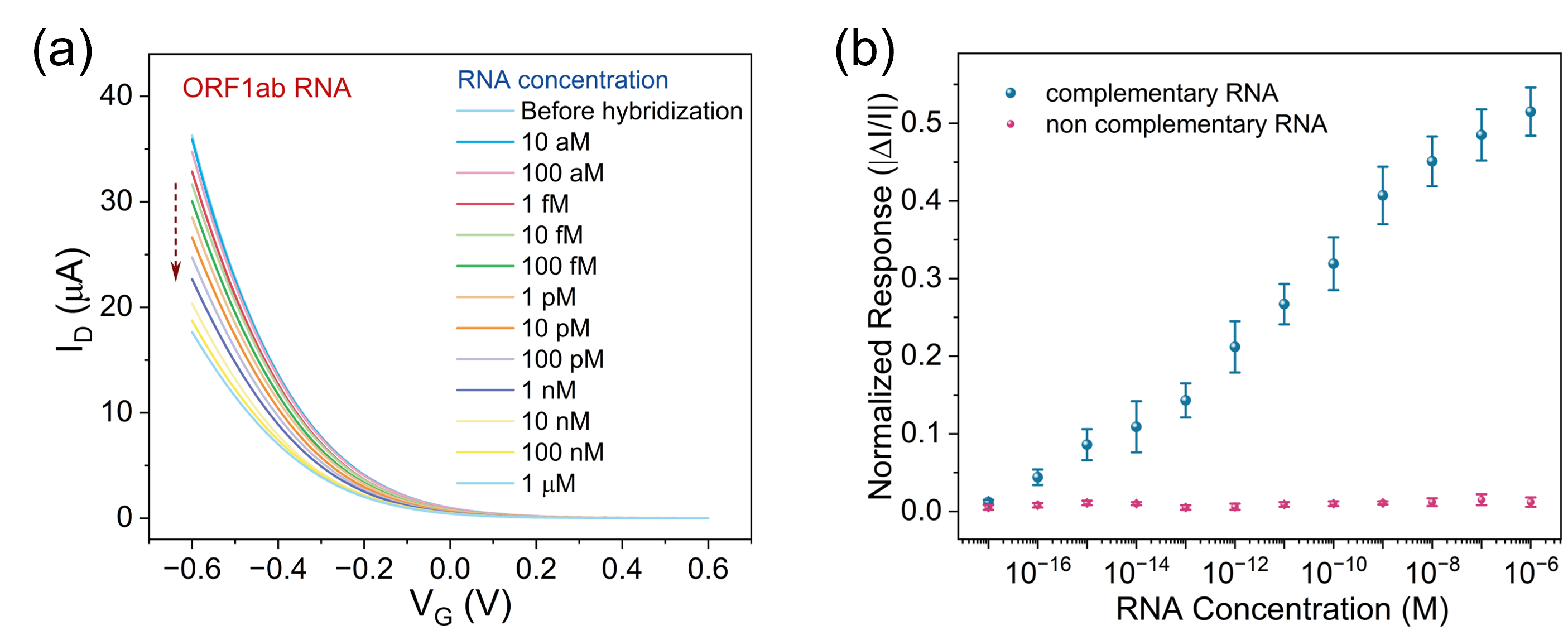


**Figure S20│ Sensing response to selected sequence of ORF1ab RNA.** (a) The transfer curve variations of OECT upon increasing concentration of ORF1ab RNA. (b) Normalized current response of the device response to complementary RNA sequence and non-complementary RNA sequence, V_DS_ = − 0.5 V.


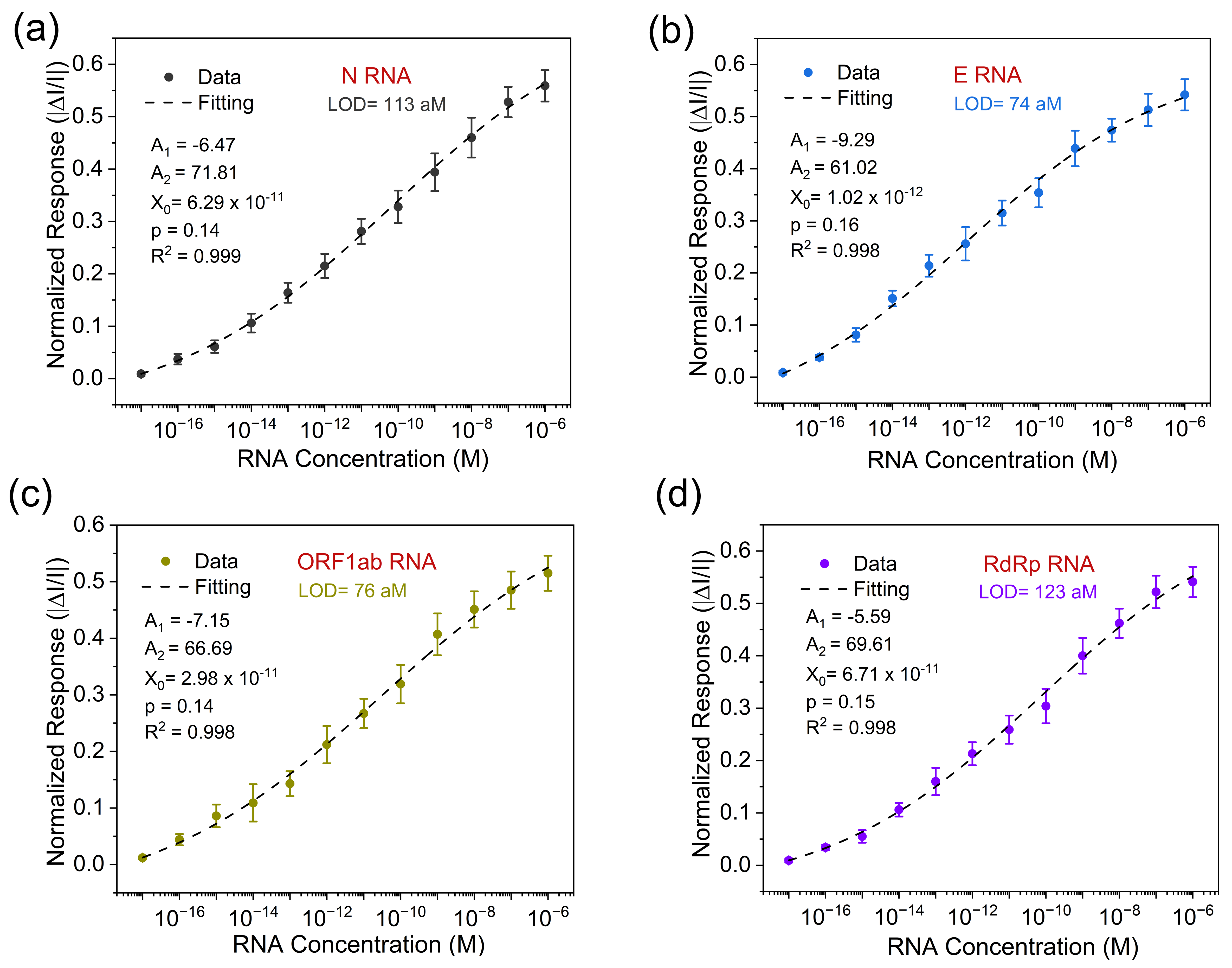


**Figure S21│** **Responses of OECTs to different RNA molecules.** Dose-response fitting curves with normalized responses and corresponding detection limits for (a) N RNA, (b) E RNA, (c) ORF1ab RNA, and (d) RdRp RNA of SARS-CoV-2.

**Table S1│** Components of the Binding Free Energy Between the Polymer and DNA-RNA hybrid.

| Receptor | DNA | | DNA-RNA | |
| --- | --- | --- | --- | --- |
| Item | Energy(kJ/mol) | Delta | Energy(kJ/mol) | Delta |
| ΔGvdw(kJ/mol) | -426.132 | 31.826 | -450.227 | 56.571 |
| ΔGele(kJ/mol) | 52.647 | 5.258 | 65.164 | 9.456 |
| ΔGPB(kJ/mol) | 169.499 | 8.403 | 140.593 | 13.511 |
| ΔGnp(kJ/mol) | -37.258 | 4.552 | -40.877 | 4.377 |
| ΔGbind(kJ/mol) | -241.244 | 6.794 | -285.347 | 18.277 |

**Table S2│**Selected sequences of SARS-CoV-2 RNA biomarkers and complementary DNA sequences.

| Name | Sequence |
| --- | --- |
| RdRp DNA | 5’-NH2-C6-G CAT CTC CTG ATG AGG TTC CAC CTG-3’ |
| RdRp RNA | 5’-CAG GUG GAA CCU CAU CAG GAG AUG C-3’ |
| E gene DNA | 5’-NH2 - C6 - CG AAG CGC AGT AAG GAT GGC TAG TGT-3’ |
| E gene RNA | 5’-ACA CUA GCC AUC CUU ACU GCG CUU CG-3’ |
| N gene DNA | 5’-NH2 - C6 - AA TCT GTC AAG CAG CAG CAA-3’ |
| N gene RNA | 5’-UUG CUG CUG CUU GAC AGA UU-3’ |
| ORF1ab DNA | 5’-NH2-C6-C CAT AAC CTT TCC ACA TAC CGC AGA CGG-3’ |
| ORF1ab RNA | 5’-CCG UCU GCG GUA UGU GGA AAG GUU AUG G-3’ |
| Single-base mismatched RNA | 5’-U AGC UUA UCA AAC UGA UGU UGA-3’ |
| Three-base mismatched RNA | 5’-U AGC GUA UCA AAC UGA CGU UGA-3’ |
| Non-complementary RNA | 5’-U UGU ACU ACA CAA AAG UAC UG-3’ |

**Table S3│**The detection limit and bio-recognition sites of OECT -based DNA/RNA sensors.

| Analytes | Limit of detection | Bio-reorganization sites | Reference |
| --- | --- | --- | --- |
| DNA | 100 fM | Gate (Carbon) | ^[4]^ |
| DNA (SA) | 81 aM | Gate (Au) | ^[5]^ |
| DNA | 0.1 fM | Gate (PEDOT: PSS) | ^[6]^ |
| DNA | 1 fM | Gate (ITO/ CdS QDs) | ^[7]^ |
| miRNA | 0.53 fM | Gate (ITO) | ^[8]^ |
| miRNA | 10 fM | Gate (Au) | ^[9]^ |
| miRNA | 0.12 fM | Gate (MOFs/TiO_2_) | ^[10]^ |
| miRNA | 5.5 fM | Gate (Au) | ^[11]^ |
| miRNA | 2 pM | Gate (Au NPs/Au) | ^[12]^ |
| miRNA | 1 pM | Gate (CdS QDs/TiO_2_) | ^[13]^ |
| miRNA | 35 aM | Channel (p(g2T-TT)) | This work |

**References:**

[1] J. R. Arias-Gonzalez,“Single-molecule portrait of DNA and RNA double helices,” *Integrative Biology* 6 (2014): 904-925, <https://doi.org/10.1039/c4ib00163j>.

[2] N. Nakatsuka, K.-A. Yang, J. M. Abendroth, et al.,“Aptamer-field-effect transistors overcome Debye length limitations for small-molecule sensing,” *Science* 362 (2018): 319-324, <https://doi.org/10.1126/science.aao6750>.

[3] A. Giovannitti, D. T. Sbircea, S. Inal, et al.,“Controlling the mode of operation of organic transistors through side-chain engineering,” *Proceedings of the National Academy of Sciences* 113 (2016): 12017-12022, <https://doi.org/10.1073/pnas.1608780113>.

[4] M. Sensi, G. Migatti, V. Beni, et al.,“Monitoring DNA hybridization with organic electrochemical transistors functionalized with polydopamine,” *Macromolecular Materials and Engineering* 307 (2022): 2100880, <https://doi.org/10.1002/mame.202100880>.

[5] J. Chen, D. Yang, D. Ji, et al.,“A fully automated point-of-care device using organic electrochemical transistor-enhanced CRISPR/Cas12a for amplification-free nucleic acid detection,” *Advanced Functional Materials* 35 (2025): 2420701, <https://doi.org/10.1002/mame.202100880>.

[6] X. Meng, Z. Yi, X. Liu, et al.,“Engineering 3D microtip gates of all-polymer organic electrochemical transistors for rapid femtomolar nucleic-acid-based saliva testing,” *Biosensors and Bioelectronics* 273 (2025): 117170, <https://doi.org/10.1016/j.bios.2025.117170>.

[7] J. Song, P. Lin, Y. Ruan, et al.,“Organic photo-electrochemical transistor-based biosensor: A proof-of-concept study toward highly sensitive DNA detection,” *Advanced Healthcare Materials* 7 (2018): 1800536, <https://doi.org/10.1002/adhm.201800536>.

[8] L. Zhang, L. Hou, H. H. Cai, et al.,“Cascading CRISPR/Cas and nanozyme for enhanced organic photoelectrochemical transistor detection with triple signal amplification,” *Analytical Chemistry* 96 (2024): 14283-14290, <https://doi.org/10.1021/acs.analchem.4c03220>.

[9] Y. Fu, N. X. Wang, A. N. Yang, et al.,“Ultrasensitive detection of ribonucleic acid biomarkers using portable sensing platforms based on organic electrochemical transistors,” *Analytical Chemistry* 93 (2021): 14359-14364, <https://doi.org/10.1021/acs.analchem.1c03441>.

[10] G. Gao, J. H. Chen, M. J. Jing, et al.,“Functional metal-organic frameworks for maximizing transconductance of organic photoelectrochemical transistor at zero gate bias and biological interfacing application,” *Advanced Functional Materials* 33 (2023): 2300580, <https://doi.org/10.1002/adfm.202300580>.

[11] P. Ju, Y. Y. Zhu, T. T. Jiang, et al.,“DNA intercalation makes possible superior-gain organic photoelectrochemical transistor detection,” *Biosensors and Bioelectronics* 237 (2023): 115543, <https://doi.org/10.1016/j.bios.2023.115543>.

[12] J. Peng, T. He, Y. L. Sun, et al.,“An organic electrochemical transistor for determination of microRNA21 using gold nanoparticles and a capture DNA probe,” *Microchimica Acta* 185 (2018): 1-8, <https://doi.org/10.1007/s00604-018-2944-x>.

[13] G. Gao, J. Hu, Z. Li, et al.,“Hybridization chain reaction for regulating surface capacitance of organic photoelectrochemical transistor toward sensitive miRNA detection,” *Biosensors and Bioelectronics* 209 (2022): 114224, <https://doi.org/10.1016/j.bios.2022.114224>.
